# K_ATP_ channels are necessary for glucose-dependent increases in amyloid-**β** and Alzheimer’s disease–related pathology

**DOI:** 10.1172/jci.insight.162454

**Published:** 2023-05-22

**Authors:** John Grizzanti, William R. Moritz, Morgan C. Pait, Molly Stanley, Sarah D. Kaye, Caitlin M. Carroll, Nicholas J. Constantino, Lily J. Deitelzweig, James A. Snipes, Derek Kellar, Emily E. Caesar, Ryan J. Pettit-Mee, Stephen M. Day, Jonathon P. Sens, Noelle I. Nicol, Jasmeen Dhillon, Maria S. Remedi, Drew D. Kiraly, Celeste M. Karch, Colin G. Nichols, David M. Holtzman, Shannon L. Macauley

**Affiliations:** 1 Department of Physiology and Pharmacology and; 2 Department of Internal Medicine, Wake Forest School of Medicine, Winston-Salem, North Carolina, USA.; 3 Department of Neurology, Washington University School of Medicine in St. Louis, St. Louis, Missouri, USA.; 4 Department of Biology, College of Arts and Sciences, University of Vermont, Burlington, Vermont, USA.; 5 Department of Medicine, Division of Endocrinology, Metabolism and Lipid Research;; 6 Department of Psychiatry;; 7 Hope Center for Neurological Disorders;; 8 Knight Alzheimer’s Disease Research Center, Department of Neurology; and; 9 Center for the Investigation of Membrane Excitability Diseases, Washington University School of Medicine in St. Louis, St. Louis, Missouri, USA.; 10 Alzheimer’s Disease Research Center;; 11 Center on Diabetes, Obesity and Metabolism;; 12 Center for Precision Medicine; and; 13 Cardiovascular Sciences Center, Wake Forest School of Medicine, Winston-Salem, North Carolina, USA.

**Keywords:** Aging, Neuroscience, Alzheimer disease, Glucose metabolism, Ion channels

## Abstract

Elevated blood glucose levels, or hyperglycemia, can increase brain excitability and amyloid-β (Aβ) release, offering a mechanistic link between type 2 diabetes and Alzheimer’s disease (AD). Since the cellular mechanisms governing this relationship are poorly understood, we explored whether ATP-sensitive potassium (K_ATP_) channels, which couple changes in energy availability with cellular excitability, play a role in AD pathogenesis. First, we demonstrate that K_ATP_ channel subunits Kir6.2/*KCNJ11* and SUR1/*ABCC8* were expressed on excitatory and inhibitory neurons in the human brain, and cortical expression of *KCNJ11* and *ABCC8* changed with AD pathology in humans and mice. Next, we explored whether eliminating neuronal K_ATP_ channel activity uncoupled the relationship between metabolism, excitability, and Aβ pathology in a potentially novel mouse model of cerebral amyloidosis and neuronal K_ATP_ channel ablation (i.e., amyloid precursor protein [APP]/PS1 Kir6.2^–/–^ mouse). Using both acute and chronic paradigms, we demonstrate that Kir6.2-K_ATP_ channels are metabolic sensors that regulate hyperglycemia-dependent increases in interstitial fluid levels of Aβ, amyloidogenic processing of APP, and amyloid plaque formation, which may be dependent on lactate release. These studies identify a potentially new role for Kir6.2-K_ATP_ channels in AD and suggest that pharmacological manipulation of Kir6.2-K_ATP_ channels holds therapeutic promise in reducing Aβ pathology in patients with diabetes or prediabetes.

## Graphical abstract

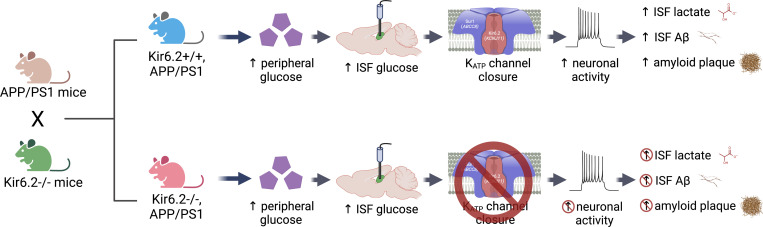

## Introduction

Epidemiological studies demonstrate that patients with type 2 diabetes mellitus (T2DM) have a 2- to 4-fold increased risk for developing Alzheimer’s disease (AD) (1–4), yet simple questions regarding how alterations in peripheral glucose metabolism affect brain function, cellular excitability, and proteostasis in AD remain relatively unexplored. People with prediabetes and those with poorly controlled diabetes experience abnormal glucose homeostasis, where elevated blood glucose levels may play a role in the development and propagation of AD-related pathology. Chronic hyperglycemia increases dementia risk (5), and individuals with elevated blood glucose levels have a higher rate of conversion from mild cognitive impairment to AD (6). Chronic hyperglycemia is also linked to alterations in brain spontaneous activity, decreased functional connectivity, and increased neuronal loss (7), suggesting that alterations in cerebral metabolism are linked to aberrant neuronal activity. This is particularly noteworthy since increased neuronal activity can stimulate amyloid-β (Aβ)/tau release, propagation, and aggregation (8–19), such that brain hyperexcitability may drive Aβ/tau accumulation. Reciprocally, the formation of amyloid plaques and neurofibrillary tangles feeds forward to cause brain hyperexcitability (19–23). Therefore, risk factors that alter neuronal excitability can have a significant impact on Aβ/tau metabolism and AD pathogenesis.

To understand how changes in peripheral metabolism influence Aβ pathology, our initial work investigated whether hyperglycemia, or elevated blood glucose levels, or hyperinsulinemia, elevated blood insulin levels, increased Aβ levels in the brain’s interstitial fluid (ISF) (12, 24). By coupling in vivo microdialysis and glucose clamp techniques, we developed a potentially novel approach to dynamically modulate systemic blood glucose and/or insulin levels while sampling proteins and metabolites within the brain’s ISF in unanesthetized, freely moving mice. We found that hyperglycemia increased Aβ production in the hippocampus through an activity-dependent mechanism, an effect that is exacerbated in mice with amyloid plaques. Interestingly, hyperinsulinemia did not have the same effect, suggesting hyperglycemia is a more potent driver of Aβ production than hyperinsulinemia. We also found a direct correlation between ISF glucose and ISF Aβ concentrations in our acute rodent studies as well as in vervet monkeys with T2DM, where blood glucose levels correlated with decreased cerebrospinal fluid (CSF) Aβ42, a biomarker of AD pathology (25). Interestingly, hyperglycemia also increased ISF lactate, a marker of neuronal activity (12, 26), suggesting alterations in cerebral metabolism are tied to brain excitability and Aβ release.

Using a pharmacological approach, we identified inward-rectifying, ATP-sensitive potassium (K_ATP_) channels as a possible mechanistic link between elevated glucose levels, neuronal excitability, and Aβ metabolism (12). K_ATP_ channels are found on the plasma membranes of excitable cells and link changes in metabolism with cellular excitability. Composed of 4 pore-forming (Kir6.1, Kir6.2) subunits and 4 sulfonylurea receptor (SUR1, SUR2A, SUR2B) binding sites, K_ATP_ channels play a role in a variety of physiological and pathological conditions (27, 28). In the pancreatic β cell, rising blood glucose levels increase intracellular ATP levels, trigger K_ATP_ channel closure, and cause insulin secretion. In the cardiovascular system, K_ATP_ channels regulate vasodilation, vasoconstriction, and vascular tone. In the brain, K_ATP_ channels are found on both neurons and glia, where increased glucose metabolism causes neuronal K_ATP_ channel closure, membrane depolarization, and increased excitability (29, 30). Thus, K_ATP_ channels act as a metabolic sensor in a wide range of tissues to effect normal physiological function.

The goal of this study was to explore the neurohumoral role of K_ATP_ channels in AD. Recent epidemiological studies demonstrate that chronic treatment with sulfonylureas, a K_ATP_ channel antagonist, can reduce the risk of developing dementia, though the mechanisms remain unknown (4, 31, 32). Therefore, we first used publicly available databases from human postmortem RNA-Seq studies to determine the cell type–specific expression of Kir6.2 (i.e., *KCNJ11*) and SUR1 (i.e., *ABCC8*) and how it changed across the AD continuum. Next, we used a genetic approach to ablate K_ATP_ channel activity in amyloid precursor protein [APP]/PS1 mice to determine 1) whether K_ATP_ channels are necessary for activity-dependent increases in ISF Aβ during acute hyperglycemia and 2) whether increased Aβ-related pathology in response to a chronic high-sucrose diet is K_ATP_ channel dependent. Deletion of the K_ATP_ channel subunit Kir6.2 (Kir6.2^–/–^) results in ablation of channel activity in neurons and abolishes glucose sensitivity. Otherwise, Kir6.2^–/–^ mice are viable and experience transient hyperinsulinemia as neonates and mild glucose intolerance as adults but thrive into adulthood (33, 34). Thus, we crossed APPswe/PSEN1dE9 (e.g., APP/PS1 mice) to Kir6.2 ^–/–^ mice. Using in vivo microdialysis and hyperglycemic clamps, we demonstrated that doubling peripheral blood glucose levels increased hippocampal ISF glucose, lactate, and Aβ levels in APP/PS1 mice with functioning K_ATP_ channels (e.g., Kir6.2^+/+^ APP/PS1 mice). Chronically, we exposed Kir6.2^+/+^ APP/PS1 mice and Kir6.2^–/–^ APP/PS1 mice to a high-sucrose diet for 6 months and demonstrated that chronic sucrose overconsumption increased both the amyloidogenic processing of APP and Aβ deposition in APP/PS1 mice with intact K_ATP_ channels. However, knocking out Kir6.2-K_ATP_ channel activity in APP/PS1 mice ablated both the acute and chronic effects. Acute hyperglycemia or chronic high-sucrose diet failed to increase ISF Aβ levels and Aβ plaque burden in Kir6.2^–/–^ APP/PS1 mice. This demonstrates that Kir6.2-containing K_ATP_ channels are necessary for hyperglycemia-dependent increases in Aβ-related pathology. Moreover, the relationship between glucose and Aβ appears to be mediated by changes in lactate production and release, which is also modulated by K_ATP_ channel activity. These data suggest that neuronal K_ATP_ channels act as metabolic sensors that couple changes in cerebral metabolism with neuronal activity and Aβ pathology. Furthermore, this work suggests that pharmacological manipulation of Kir6.2-K_ATP_ channels may hold therapeutic benefit in reducing Aβ pathology for patients with diabetes or prediabetes.

## Results

### K_ATP_ channels, composed of Kir6.2 and SUR1, are found on neurons and have altered expression in the human AD brain and rodent models of AD-related pathology.

**Figure 1:**
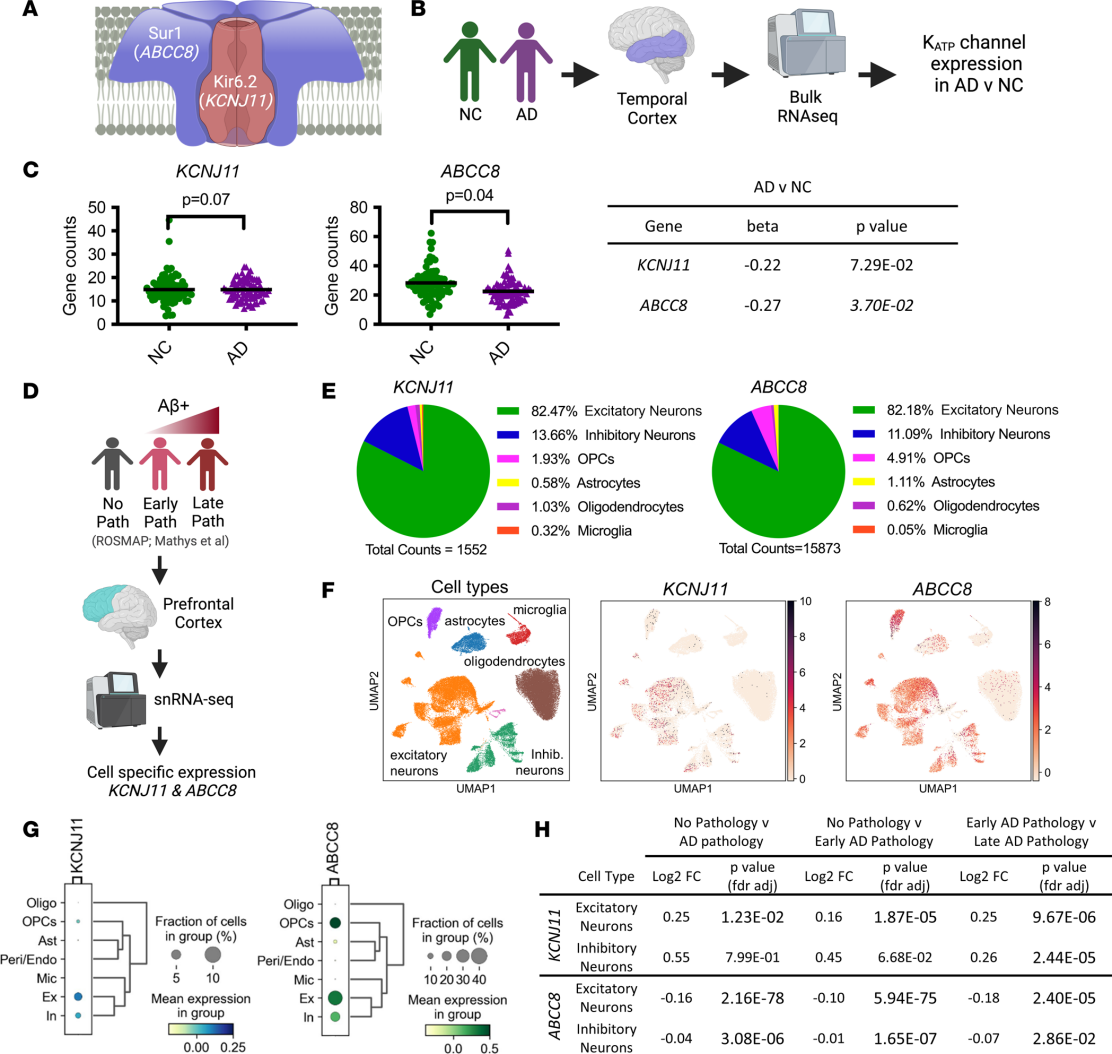
K_ATP_ channel expression in postmortem humans across the AD continuum. (**A**) K_ATP_ channels are heteroctameric, composed of 4 pore-forming subunits (Kir6.2*/KCNJ11*) and 4 sulfonylurea-binding site (Sur1*/ABCC8*) subunits. (**B**) Workflow to explore how K_ATP_ channel genes (*KCNJ11*, *ABCC8*) in the temporal cortex change because of AD-related pathology using the Mayo RNA-Seq database. (**C**) *KCNJ11* expression trends toward a decrease in AD (amyloid^+^, tau^+^), while ABCC8 is significantly reduced in AD in bulk RNA-Seq. (**D**) Workflow to explore cell type–specific changes in *KCNJ11* and *ABCC8* expression in postmortem human brains using single-nuclei RNA-Seq (snRNA-Seq) database generated by Mathys et al. (35). (**E**) *KCNJ11* and *ABCC8* expression is largely found on excitatory and inhibitory neurons (>96%) but also localized to glia, like oligodendrocyte progenitor cells (OPCs). (**F**) NC and AD samples were integrated into a single data set and clustered into cell types. Uniform manifold approximation and projection (UMAP) representation of different CNS cell types, including relative expression for *KCNJ11* and *ABCC8*. (**G**) Gene expression dot blot for *KCNJ11* and *ABCC8* demonstrating relative expression levels in each cell type. (**H**) Comparing postmortem brains with no AD pathology (*n* = 24), early AD pathology (*n* = 15), and late AD pathology (*n* = 9), *KCNJ11* expression is increased on excitatory neurons with early and late pathology while *KCNJ11* expression is increased on inhibitory neurons at a late stage of disease. *ABCC8*, the binding partner of *KCNJ11*, is reduced in inhibitory and excitatory neurons across the AD continuum. All data represented as means (statistically analyzed using Student’s *t* test) ± SEM. *P < 0.05.

*KCNJ11* encodes the pore-forming subunit Kir6.2, and *ABCC8* encodes the regulatory sulfonylurea-binding subunit SUR1. Kir6.2 and SUR1 heteromultimerize to form neurohumoral K_ATP_ channels (Figure 1A). To assess K_ATP_ channel gene expression in AD brain tissue, we analyzed gene expression of *KCNJ11* and *ABCC8* in 2 publicly available data sets (Figure 1, B–H). First, we analyzed gene expression data from the temporal cortex of normal control (NC; *n* = 80) and AD (amyloid^+^, tau^+^; *n* = 82) brains in the Mayo Clinic Brain Bank (Mayo) RNA-Seq study from the Accelerating Medicines Partnership – Alzheimer’s Disease (AMP-AD) portal (Figure 1B). In AD, *KCNJ11* expression trended toward a decrease (β = –0.2150, *P* < 7.29 × 10^–2^), while *ABCC8* was significantly reduced in AD brains (β = –0.2669, *P* < 3.7 × 10^–2^, respectively). Next, we leveraged a publicly available single-nuclei RNA-Seq (snRNA-Seq) database from postmortem human prefrontal cortex (35) to explore the cellular localization and expression profile of *KCNJ11* and *ABCC8* across the AD continuum (Figure 1D). First, both *KCNJ11* and *ABCC8* were predominately localized to excitatory neurons (>80%) or inhibitory neurons (10%–12%), and to a lesser degree, oligodendrocyte progenitor cells (2%–4%), oligodendrocytes (~1%), astrocytes (<2%), or microglia (<1%; Figure 1E). Across the AD continuum, *KCNJ11* expression was increased in early and late AD in excitatory neurons, while *KCNJ11* expression was increased in late-stage AD in inhibitory neurons (Figure 1, E–G). In contrast, *ABCC8* expression was decreased in both excitatory and inhibitory neurons in early and late AD compared with individuals with no pathology (Figure 1H). There was no change in *KCNJ11* expression in glia across the AD continuum (data not shown).

**Figure 2:**
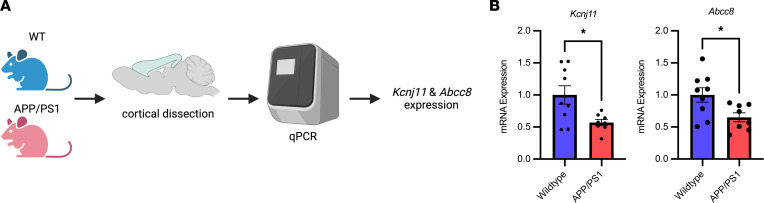
K_ATP_ channel expression changes in APP/PS1 mice in response to Aβ pathology. (**A**) Workflow for exploring *Kcnj11* and *Abcc8* expression in the mouse cortex of APP/PS1 and WT mice. (**B**) Decreased *Kcnj11* and *Abcc8* expression in 9-month APP/PS1 cortex compared with 9-month WT mice. All data represented as means (statistically analyzed using Student’s *t* test) ± SEM. *P < 0.05.

Last, we performed quantitative PCR (qPCR) on the cortex of 9-month-old APP/PS1 and wild-type controls (Figure 2A). *Kcnj11* and *Abcc8* expression was decreased in APP/PS1 mice compared with wild-type controls (Figure 2B). This demonstrates that Kir6.2-K_ATP_ channels are localized to both excitatory and inhibitory neurons. It also demonstrates that *KCNJ11* and *ABCC8* expression is altered across the AD continuum in humans and mice, where these changes appear to be dependent on the stage of AD and brain region sampled. It also suggests that the stoichiometry of the Kir6.2-K_ATP_ channel subunits is altered (e.g., *KCNJ11* vs. *ABCC8*) with AD-related pathology, which could impact its role as a metabolic sensor in AD.

### Kir6.2 deficiency differentially influences peripheral and cerebral metabolism in APP/PS1 mice.

**Figure 3:**
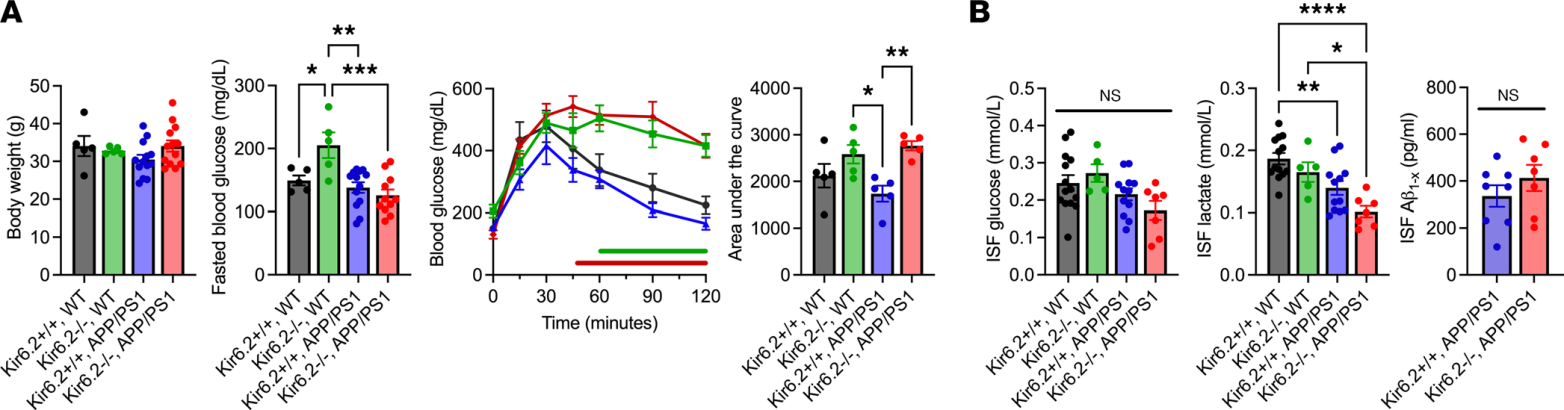
Kir6.2^–/–^ differentially affects peripheral and cerebral metabolism at baseline in WT and APP/PS1 mice at 3–4 months of age. (**A**) No difference in body weight was observed across genotypes. Kir6.2^–/–^ WT mice had increased fasted blood glucose, but this effect was lost in Kir6.2^–/–^ APP/PS1 mice (*n* = 5–13/group; *P* = 0.0006). Both Kir6.2^–/–^ WT and Kir6.2^–/–^ APP/PS1 mice were glucose intolerant when compared with Kir6.2^+/+^ APP/PS1 mice. (**B**) Steady-state levels of ISF glucose trended toward a decrease in Kir6.2^–/–^ APP/PS1 mice, but the effect was not significant (*P* < 0.06; *n* = 5–13/group). ISF lactate levels were decreased in APP/PS1 mice compared with WT controls, an effect exacerbated by Kir6.2^–/–^ (*P* < 0.0001; *n* = 5–14). There was no difference in steady-state ISF Aβ levels (*n* = 7–9 group). One-way ANOVA or 2-way ANOVA with Tukey’s post hoc tests or Student’s *t* tests were used to determine significance (*P* < 0.05). All data represented as means ± SEM. *P < 0.05, **P < 0.01, ***P < 0.001, ****P < 0.0001.

To explore the role of Kir6.2-dependent K_ATP_ channels in AD-related pathology, we crossed mice deficient in Kir6.2 (i.e., Kir6.2^–/–^ mice) (33) with APPswe/PSEN1dE9 (i.e., APP/PS1) (36) mice to generate mice overexpressing Aβ that were also deficient in neuronal K_ATP_ channel activity (i.e., Kir6.2^–/–^ APP/PS1). This model could then be used to explore whether neuronal K_ATP_ channels act as metabolic sensors, linking changes in cerebral metabolism with neuronal excitability and Aβ release. First, we characterized how Kir6.2-K_ATP_ channel deficiency affected peripheral and cerebral metabolism in 5-month-old male APP/PS1 mice and WT mice with or without Kir6.2^–/–^ (Figure 3A). There was no difference in body weight between groups; however, alterations in peripheral and cerebral glucose homeostasis were observed. Kir6.2^–/–^ WT mice had elevated blood glucose levels compared with all other groups (*P* = 0.0006). Additionally, Kir6.2^–/–^ WT (*P* = 0.0278) and Kir6.2^–/–^ APP/PS1 (*P* = 0.0071) mice were glucose intolerant compared with Kir6.2^+/+^ APP/PS1 mice. Using in vivo microdialysis, we also assessed steady-state ISF levels of glucose, lactate, and Aβ in the hippocampus. Kir6.2^–/–^ APP/PS1 mice showed a trending decrease in ISF glucose levels compared with Kir6.2^+/+^ WT (*P* = 0.1029) and Kir6.2^–/–^ WT (*P* = 0.0707) mice (Figure 3B), but this effect was not significant (*P* = 0.06). Similarly, APP/PS1 mice, with or without Kir6.2-K_ATP_ channels, had reduced ISF lactate levels compared with WT mice (*P* < 0.0001). ISF Aβ levels between Kir6.2^+/+^ APP/PS1 and. Kir6.2^–/–^ APP/PS1 mice did not differ at 5 months of age (Figure 3B). Together, these data suggest that Kir6.2^–/–^ WT and Kir6.2^–/–^ APP/PS1 mice have impaired peripheral glucose metabolism, while both Kir6.2^–/–^ APP/PS1 and Kir6.2^+/+^ APP/PS1 have changes in cerebral metabolism.

### Kir6.2-K_ATP_ channels link acute changes in brain glucose with ISF Aβ and lactate levels.

**Figure 4:**
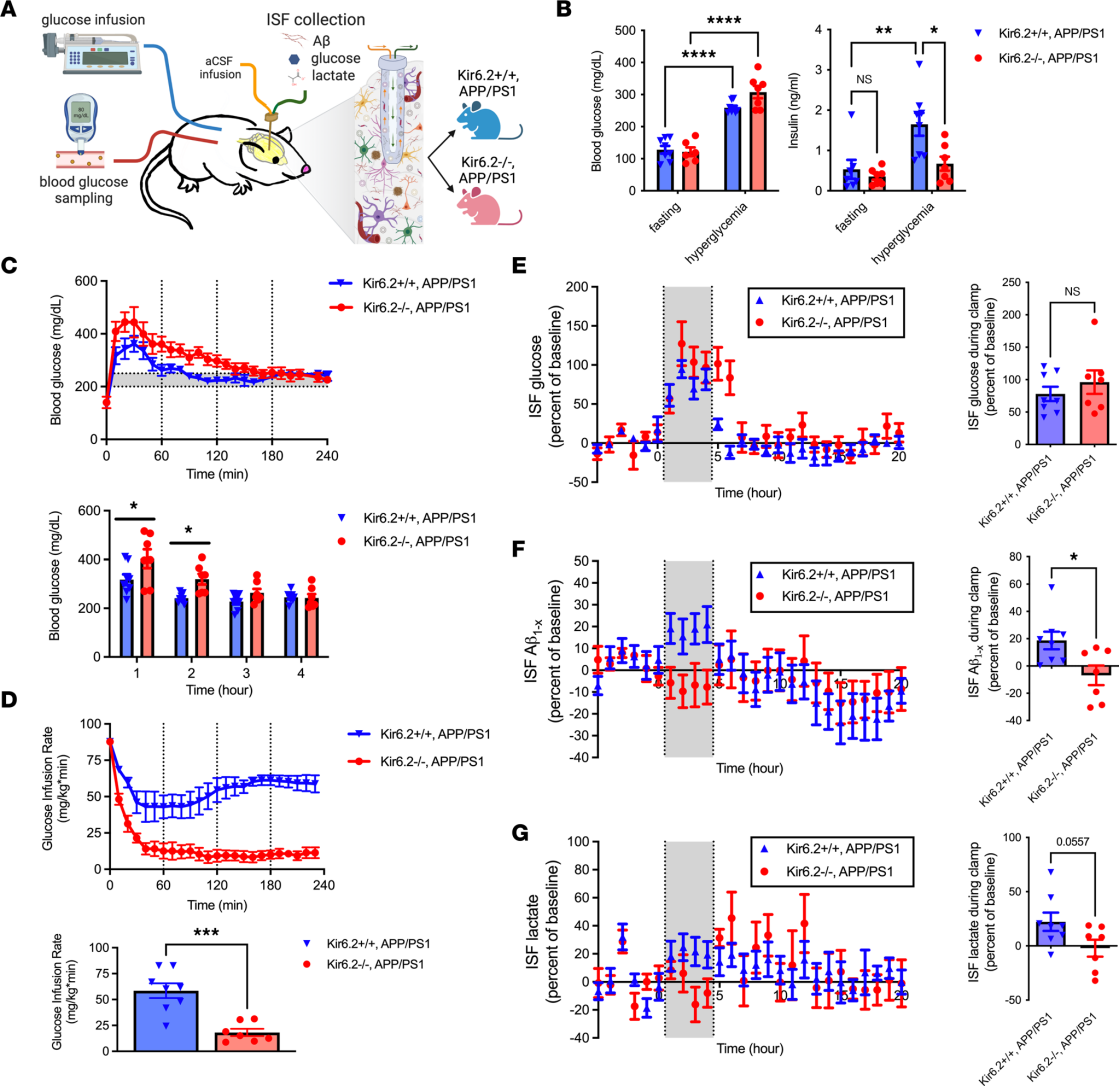
Kir6.2^–/–^ alters the peripheral and brain response to acute hyperglycemia in APP/PS1 mice, including ISF Aβ levels. (**A**) Experimental approach where hyperglycemic clamps are paired with in vivo microdialysis to assess acute changes in ISF levels of Aβ, glucose, and lactate during hyperglycemia in male Kir6.2^+/+^ APP/PS1 and Kir6.2^–/–^ APP/PS1 mice (*n* = 7–8/group). aCSF, artificial cerebrospinal fluid. (**B**) Hyperglycemia caused a 1.1-fold and 1.5-fold increase in blood glucose levels in Kir6.2^+/+^ APP/PS1 or Kir6.2^–/–^ APP/PS1 mice, respectively. While no difference in fasting insulin levels existed at baseline, hyperglycemia increased insulin levels in the Kir6.2^+/+^ APP/PS1 mice 2.1-fold while insulin levels did not change in Kir6.2^–/–^ APP/PS1 mice. (**C**) Blood glucose levels were higher during the first 2 hours of the clamp in Kir6.2^–/–^ APP/PS1 mice compared with Kir6.2^+/+^ APP/PS1 mice. (**D**) There was a 3.2-fold decrease in the glucose infusion rate for the Kir6.2^–/–^ APP/PS1 mice compared with Kir6.2^+/+^ APP/PS1 mice due to an attenuated insulin response. (**E**) ISF glucose levels increased during the clamp to comparable levels in Kir6.2^–/–^ APP/PS1 and Kir6.2^+/+^ APP/PS1 mice (*n* = 7–8 mice/group). (**F**) During hyperglycemia, no increase in ISF Aβ was observed in Kir6.2^–/–^ APP/PS1 mice despite a 19% increase in ISF Aβ in Kir6.2^+/+^ APP/PS1 mice. (**G**) Similarly, ISF lactate increased in Kir6.2^+/+^ APP/PS1 mice by 22% while no increase was observed in Kir6.2^–/–^ APP/PS1 mice. One-way ANOVA or 2-way ANOVA with Tukey’s post hoc tests or Student’s *t* tests were used to determine significance (*P* < 0.05). All data represented as means (statistically analyzed using Student’s *t* test) ± SEM. *P < 0.05, **P < 0.01, ***P < 0.001, ****P < 0.0001.

We previously combined hyperglycemic clamps and in vivo microdialysis as a tool to investigate how dynamic changes in blood glucose levels alter metabolites and proteins in real time in the hippocampal ISF from unrestrained, freely moving mice (12, 37). By coupling this approach with our Kir6.2^–/–^ APP/PS1 mouse model, we explored whether neuronal K_ATP_ channels are necessary for glucose-dependent increases in hippocampal ISF Aβ and ISF lactate (Figure 4A). The target blood glucose range for hyperglycemic clamps was 250–300 mg/dL, resulting in a 100%–150% increase of blood glucose levels relative to fasted baseline in Kir6.2^+/+^ APP/PS1 (*P* < 0.0001) and Kir6.2^–/–^ APP/PS1 mice (*P* < 0.0001; Figure 4B). In response to hyperglycemia, Kir6.2^+/+^ APP/PS1 mice also showed a 210% increase in plasma insulin compared with fasting levels (*P* = 0.002), while no change on plasma insulin levels was observed in Kir6.2^–/–^ APP/PS1 mice (*P* = 0.8976; Figure 4B). This is due to the lack of Kir6.2-K_ATP_ channels on pancreatic β cells, which perturbs insulin secretion (38). Reduced insulin secretion in Kir6.2^–/–^ APP/PS1 mice was also accompanied by higher blood glucose levels during the first 2 hours of the clamp (Figure 4C) and a 68% reduction in glucose infusion rates, a metric of insulin responsiveness to hyperglycemia, compared with Kir6.2^+/+^ APP/PS1 mice during the hyperglycemic clamp (*P* = 0.0003; Figure 4D). Regardless, blood glucose levels in Kir6.2^+/+^ APP/PS1 and Kir6.2^–/–^ APP/PS1 mice were comparable during the 4-hour hyperglycemic clamp despite the differential effect on plasma insulin levels and the insulin response.

During the 4-hour hyperglycemic clamp, hippocampal ISF glucose levels increased comparably in Kir6.2^+/+^ APP/PS1 and Kir6.2^–/–^ APP/PS1 mice (Figure 4E). This was associated with an approximately 20% increase in ISF Aβ in Kir6.2^+/+^ APP/PS1 mice, but there was no increase in ISF Aβ levels in Kir6.2^–/–^ APP/PS1 mice (Figure 4F). ISF lactate is the metabolic end product of glycolysis and used as a marker of neuronal activity, where it covaries with increased EEG amplitude and ISF Aβ levels (9, 12, 14, 26, 39). In Kir6.2^+/+^ APP/PS1 mice, ISF lactate increased by approximately 22% ± 8.4% during hyperglycemia, while no increase in ISF lactate was observed in Kir6.2^–/–^ APP/PS1 mice (Figure 4G). Together, these data demonstrate that Kir6.2-K_ATP_ channels act as metabolic sensors to couple elevated ISF glucose with elevated ISF Aβ and ISF lactate.

**Figure 5:**
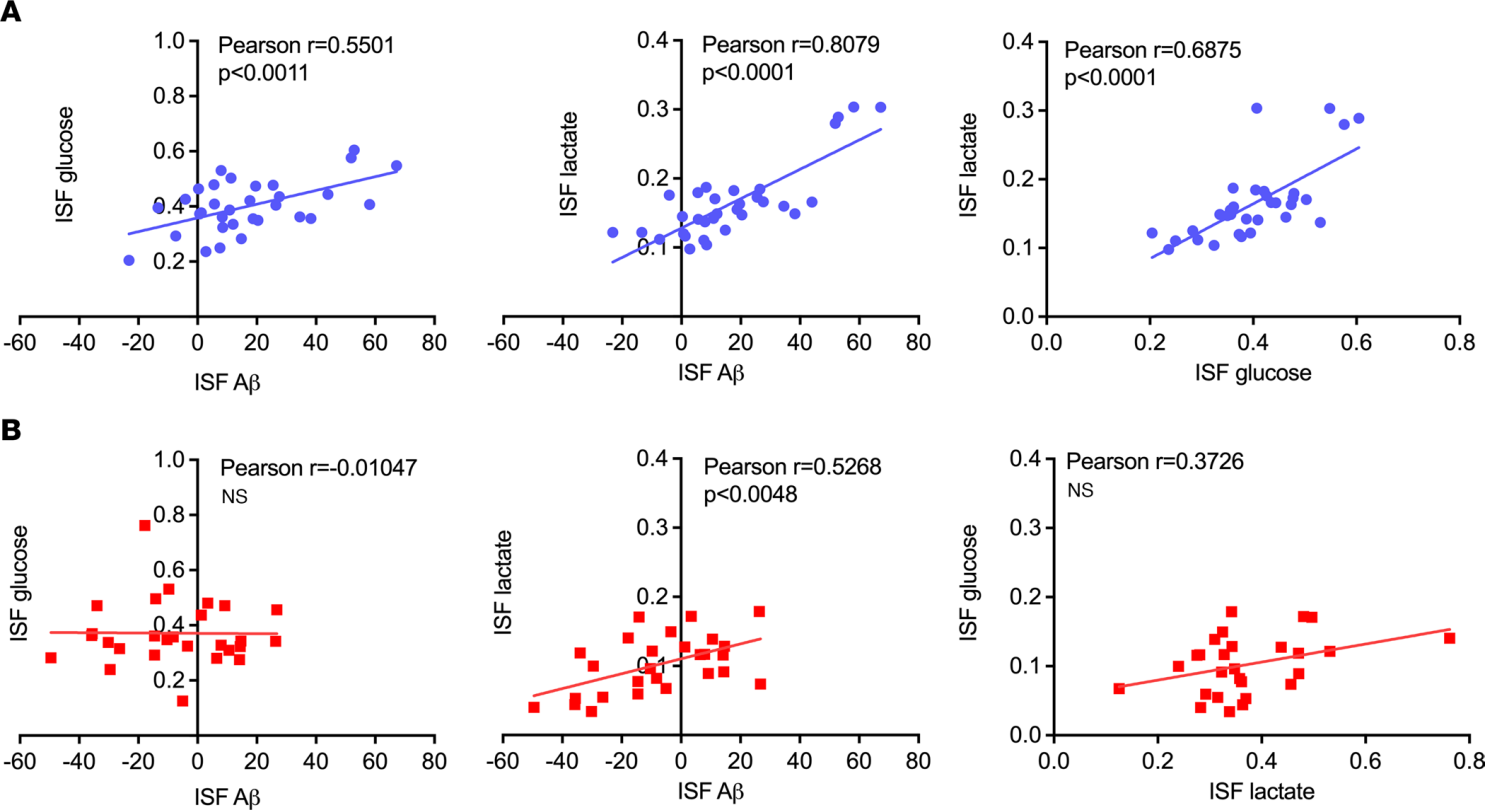
Correlations between ISF glucose, ISF Aβ, and ISF lactate in APP/PS1 mice with or without Kir6.2-K_ATP_ channels during hyperglycemic clamps. (**A**) In male Kir6.2^+/+^ APP/PS1 mice (shown in blue), ISF glucose, ISF Aβ, and ISF lactate display a positive correlation (*n* = 20). (**B**) Conversely, in Kir6.2^–/–^ APP/PS1 mice (red), ISF glucose is no longer correlated with ISF Aβ or ISF lactate. All relationships were analyzed using Pearson’s *r* correlation analysis.

Pearson’s correlations were performed on hippocampal ISF glucose, lactate, and Aβ levels during hyperglycemia to further explore the effects of K_ATP_ channel deletion on these relationships. ISF Aβ levels showed strong, moderate correlations (Figure 5A) with glucose (*r* = 0.5501, *P* = 0.0011) and lactate (*r* = 0.8079, *P* < 0.0001) in Kir6.2^+/+^ APP/PS1 mice. Similarly, ISF glucose and lactate also displayed a strong, positive correlation in Kir6.2^+/+^ APP/PS1 mice (*r* = 0.6875, *P* < 0.0001), demonstrating a positive relationship among energy demand, neuronal activity, and Aβ release. The relationships among ISF glucose, Aβ, and lactate were uncoupled in Kir6.2^–/–^ APP/PS1 mice. The correlations between ISF glucose and ISF Aβ as well as ISF glucose and ISF lactate were lost in Kir6.2^–/–^ APP/PS1 mice (Figure 5B), while a correlation between ISF Aβ and lactate (*r* = 0.5268, *P* = 0.0048) persisted but only when ISF Aβ decreased (Figure 5B). This suggests that Kir6.2-dependent K_ATP_ channels act as metabolic sensors and are necessary for hyperglycemia-dependent increases in both hippocampal ISF Aβ and ISF lactate. It also suggests that Kir6.2-dependent K_ATP_ channels couple ISF glucose with ISF lactate, which may be necessary for increased ISF Aβ levels.

To verify that hyperglycemia-dependent increases in ISF Aβ were Kir6.2 dependent, we explored whether inhibition of Kir6.1-containing K_ATP_ channels, which are predominantly localized to the vasculature, could directly modulate ISF Aβ levels. APP/PS1 mice deficient in Kir6.1 (Kir6.1^–/–^ APP/PS1) or Kir6.2 (Kir6.2^–/–^ APP/PS1) received a hippocampal infusion of K_ATP_ channel antagonist glibenclamide via reverse microdialysis. Glibenclamide infusion raised ISF Aβ levels in both Kir6.2^+/+^ APP/PS1 and Kir6.1^–/–^ APP/PS1 mice by 29% ± 9.1% and 24% ± 6.7%, respectively, but not in Kir6.2^–/–^ APP/PS1 mice (Supplemental Figure 1, A and B; supplemental material available online with this article; https://doi.org/10.1172/jci.insight.162454DS1). This suggests that the changes in ISF Aβ during hyperglycemia are dependent on Kir6.2-containing K_ATP_ channels, not Kir6.1-K_ATP_ channels. We also explored whether Kir6.2^–/–^ shifted the localization of Kir6.1 subunits in the hippocampus (Supplemental Figure 1, C and D). No change in Kir6.1 localization was observed in Kir6.2^–/–^ mice. Together, this suggests that Kir6.2- K_ATP_ channels are responsible for glucose-dependent increases in ISF Aβ in the brain.

### Kir6.2-K_ATP_ channels couple chronic changes in peripheral glucose levels with Aβ pathology.

**Figure 6:**
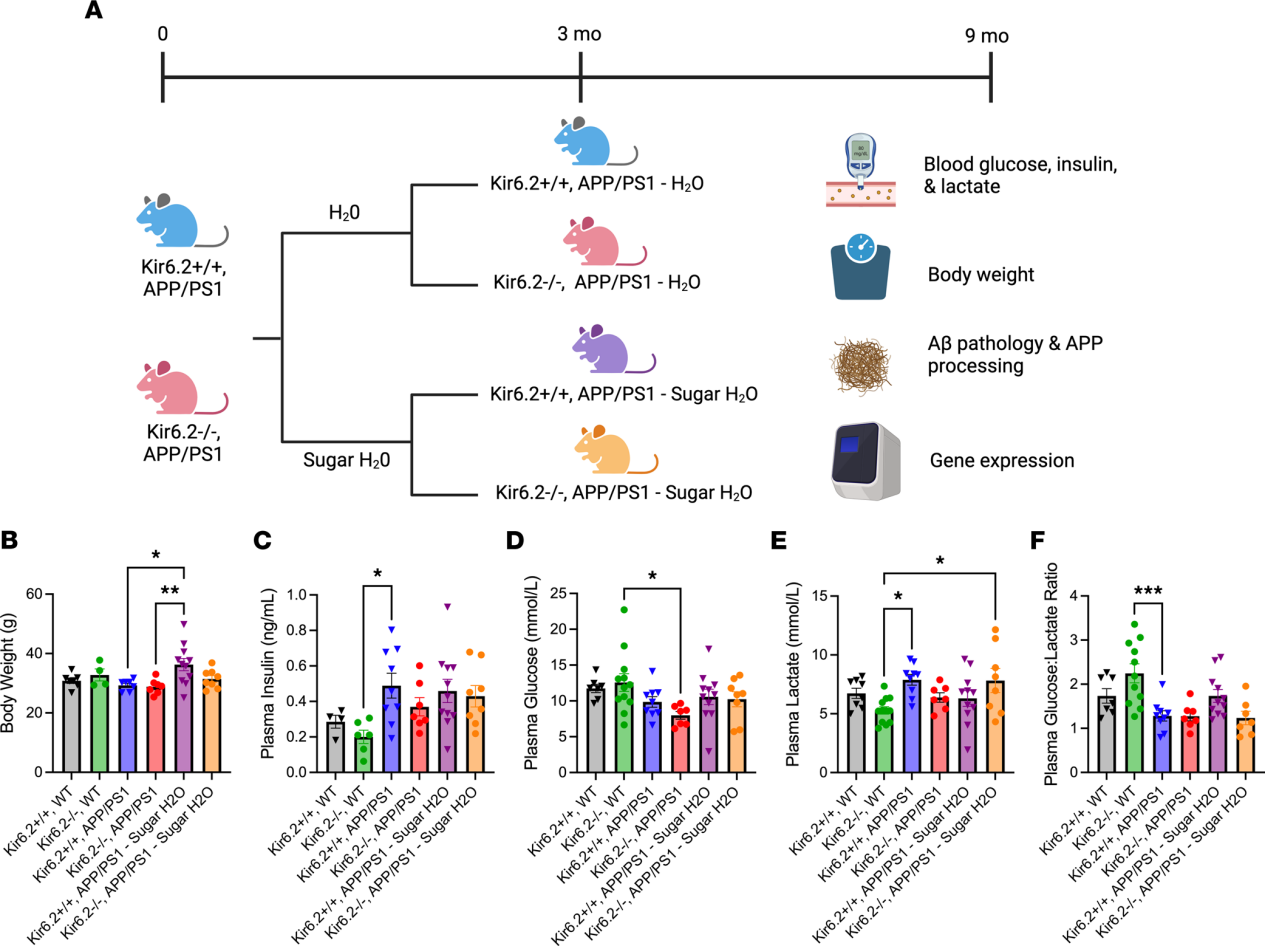
Chronic sugar exposure differentially affects peripheral metabolism in Kir6.2^+/+^ APP/PS1 and Kir6.2^–/–^ APP/PS1 mice. (**A**) Schematic of experimental approach where female Kir6.2^+/+^ APP/PS1 and Kir6.2^–/–^ APP/PS1 mice were fed regular drinking water or high-glucose, high-fructose drinking water for 6 months (*n* = 7–11/group). (**B**) Kir6.2^+/+^ APP/PS1 mice on a high-sucrose diet had increased body weight compared with Kir6.2^+/+^ APP/PS1 mice (*P* = 0.0169) and Kir6.2^–/–^ APP/PS1 mice (*P* = 0.0091). (**C**) Plasma insulin levels were decreased in Kir6.2^–/–^ WT mice compared with Kir6.2^+/+^ APP/PS1 mice (*P* = 0.0363). (**D**) Plasma glucose levels were increased in Kir6.2^–/–^ WT mice compared with Kir6.2^–/–^ APP/PS1 mice (*P* = 0.0335). (**E**) Plasma lactate levels were reduced in Kir6.2^–/–^ WT mice compared with Kir6.2^+/+^ APP/PS1 (*P* = 0.0182) and Kir6.2^–/–^ APP/PS1 – sugar H_2_O mice (*P* = 0.0305). (**F**) Kir6.2^–/–^, WT mice had an increased plasma glucose/lactate ratio compared with Kir6.2^+/+^ APP/PS1 mice (*P* = 0.0003). Together, these data demonstrate that Kir6.2^–/–^ WT mice have lower circulating insulin levels, which results in aberrant glucose metabolism. All relationships were analyzed via 1-way ANOVA with Tukey’s post hoc analysis. All data were analyzed via 1-way ANOVA and represented as means ± SEM. *P < 0.05, **P < 0.01, ***P < 0.001.

Beginning at 3 months of age, Kir6.2^+/+^ APP/PS1 and Kir6.2^–/–^ APP/PS1 mice were exposed to high-sucrose drinking water (e.g., sugar H_2_O) or normal drinking water for 6 months to assess the role of Kir6.2-dependent K_ATP_ channels in glucose-dependent amyloid plaque formation and AD-related pathology (*n* = 8–11 mice/group; Figure 6A). Prior to sacrifice, terminal body weights, plasma insulin, plasma glucose, and plasma lactate measurements were performed to determine the effects of a high-sucrose diet on peripheral metabolism and its relationship to AD-related pathology.

At 9 months of age, Kir6.2^+/+^ APP/PS1 mice that consumed sugar H_2_O for 6 months weighed approximately 20% more than Kir6.2^+/+^ APP/PS1 controls (*P* = 0.0169; Figure 6B). While genotype and diet had modest effects on plasma insulin, glucose, and lactate levels, some patterns did emerge. Kir6.2^–/–^ mice had decreased plasma insulin, increased plasma glucose, decreased plasma lactate, and increased plasma glucose/lactate ratio (Figure 6, C–F), a metric of nonoxidative/oxidative glucose metabolism (40–43), when compared with APP/PS1 mice. These effects were abolished in Kir6.2^–/–^ mice on either an APP/PS1 background or fed a high-sugar diet, suggesting amyloid pathology has a strong influence on peripheral metabolism.

**Figure 7:**
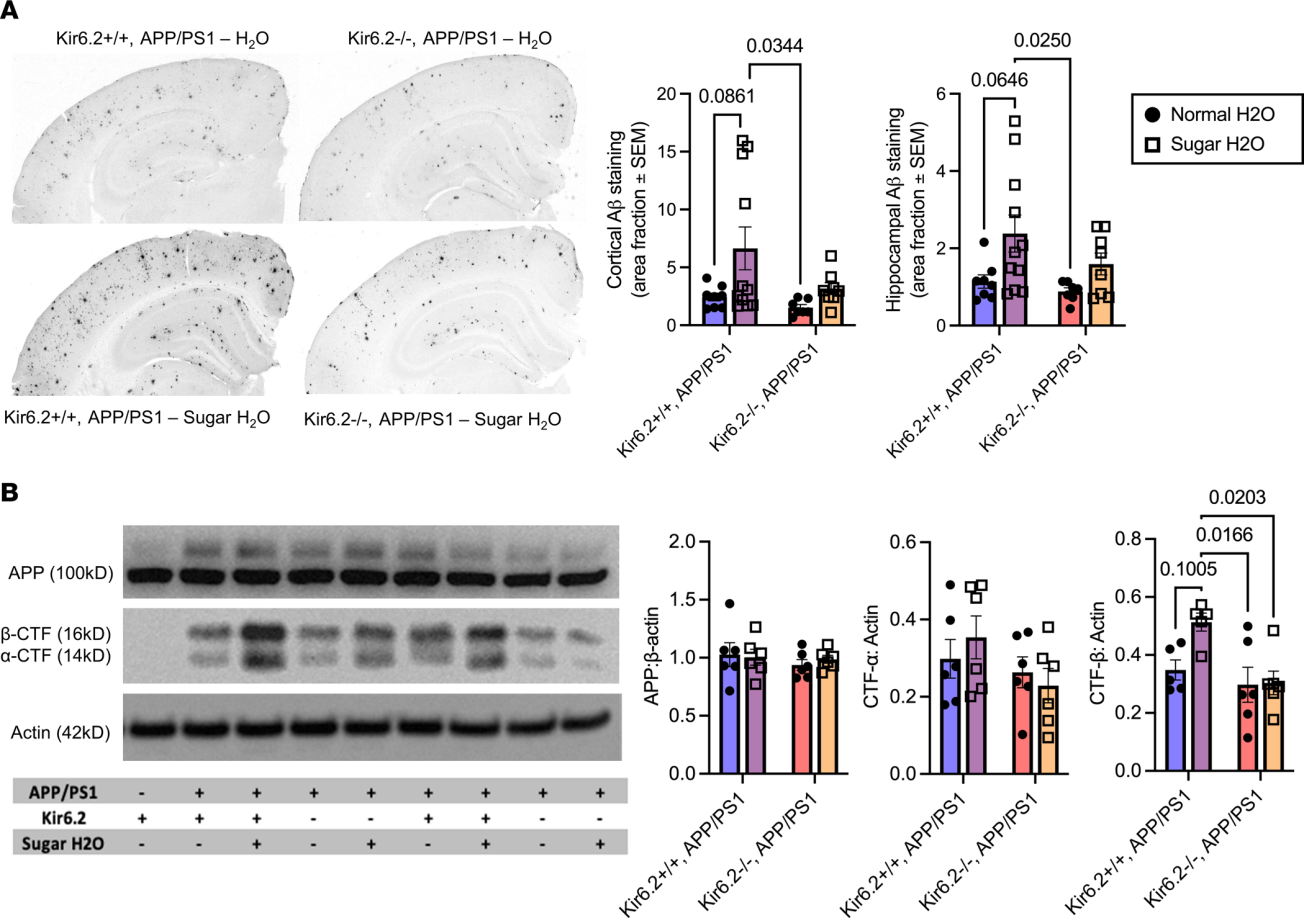
APP/PS1 mice fed a high-sugar diet had increased Aβ deposition and amyloidogenic processing of APP. (**A**) Representative images of Aβ deposition in Kir6.2^+/+^ APP/PS1 and Kir6.2^–/–^ APP/PS1 mice fed a normal (black circles) or high-sugar diet (open squares; *n* = 7–11/group). Aβ deposition was increased in both the cortex (main effect of diet, *P* = 0.0293) and the hippocampus (main effect of diet, *P* = 0.0104) of the Kir6.2^+/+^ APP/PS1 mice fed a high-sugar diet but not in Kir6.2^–/–^ APP/PS1 mice fed a high-sugar diet via 2-way repeated measures ANOVAs and Tukey’s post hoc analyses (represented as mean ± SEM). Original magnification, ×3. (**B**) Western blot analysis for APP and the C-terminal fragment (CTF) showed no difference in the expression of full-length APP or CTF-α between any groups, but there was an increase in CTF-β in Kir6.2^+/+^ APP/PS1 mice fed a high-sugar diet (black circles; *n* = 6/group) compared with Kir6.2^–/–^ APP/PS1 – H_2_O (*P* = 0.0166) and Kir6.2^–/–^ APP/PS1 – sugar H_2_O mice (*P* = 0.0203) via 2-way repeated measures ANOVAs and Tukey’s post hoc analyses (represented as mean ± SEM).

To evaluate how a high-sugar diet affected Aβ-related pathology in Kir6.2^+/+^ APP/PS1 and Kir6.2^–/–^ APP/PS1 mice, we quantified Aβ deposition via immunohistochemistry and APP processing via Western blot analysis (Figure 7). First, 50 μm sections were immunostained with the monoclonal Aβ antibody, HJ3.4B, to identify Aβ deposition within the cortex and hippocampus. Percentage area coverage in both regions was quantified for the following 4 groups: 1) Kir6.2^+/+^ APP/PS1 – H_2_O mice, 2) Kir6.2^–/–^ APP/PS1 – H_2_O mice, 3) Kir6.2^+/+^ APP/PS1 – sugar H_2_O mice, and 4) Kir6.2^–/–^ APP/PS1 – sugar H_2_O mice (Figure 7A). Genotype alone did not impact Aβ deposition in the APP/PS1 mice, where Kir6.2^+/+^ APP/PS1 mice and Kir6.2^–/–^ APP/PS1 mice fed normal drinking water had comparable levels of Aβ deposition in both cortex and hippocampus. However, Kir6.2^+/+^ APP/PS1 mice given high-sugar H_2_O had 2–3 times more Aβ plaques in the cortex (main effect of diet, *P* = 0.0293) and hippocampus (main effect of diet, *P* = 0.0104) compared with Kir6.2^+/+^ APP/PS1 mice or Kir6.2^–/–^ APP/PS1 mice fed a normal diet (Figure 7A). Conversely, high-sucrose diet did not increase Aβ deposition in Kir6.2^–/–^ APP/PS1 mice. This shows that chronic overconsumption of sucrose increased Aβ deposition in APP/PS1 mice but not in mice lacking Kir6.2-K_ATP_ channel activity (Figure 6A). This suggests that Kir6.2-K_ATP_ channels are necessary for sucrose-dependent increases in Aβ pathology.

To determine whether chronic sucrose consumption altered APP metabolism, APP processing was analyzed through measurement of total APP as well as the protein levels of 2 key APP metabolites, CTF-α and CTF-β (Figure 7B). There were no changes in the levels of APP or CTF-α due to genotype or consumption of sugar H_2_O (Figure 7B). Importantly, there was an increase in CTF-β in Kir6.2^+/+^ APP/PS1 mice fed a high-sugar diet compared with Kir6.2^–/–^ APP/PS1 – H_2_O (*P* = 0.0166) and Kir6.2^–/–^ APP/PS1 – sugar H_2_O mice (*P* = 0.0203), suggesting that chronic sugar consumption increased the amyloidogenic processing of APP to produce more CTF-β and Aβ in Kir6.2^+/+^ APP/PS1 mice. Together, these data suggest that Kir6.2-containing K_ATP_ channels were necessary for increased Aβ deposition (Figure 7A), amyloidogenic processing of APP, and Aβ production (Figure 7B) in Kir6.2^+/+^ APP/PS1 mice following chronic sucrose exposure, yet the effect was lost with Kir6.2-K_ATP_ channel deletion.

**Figure 8:**
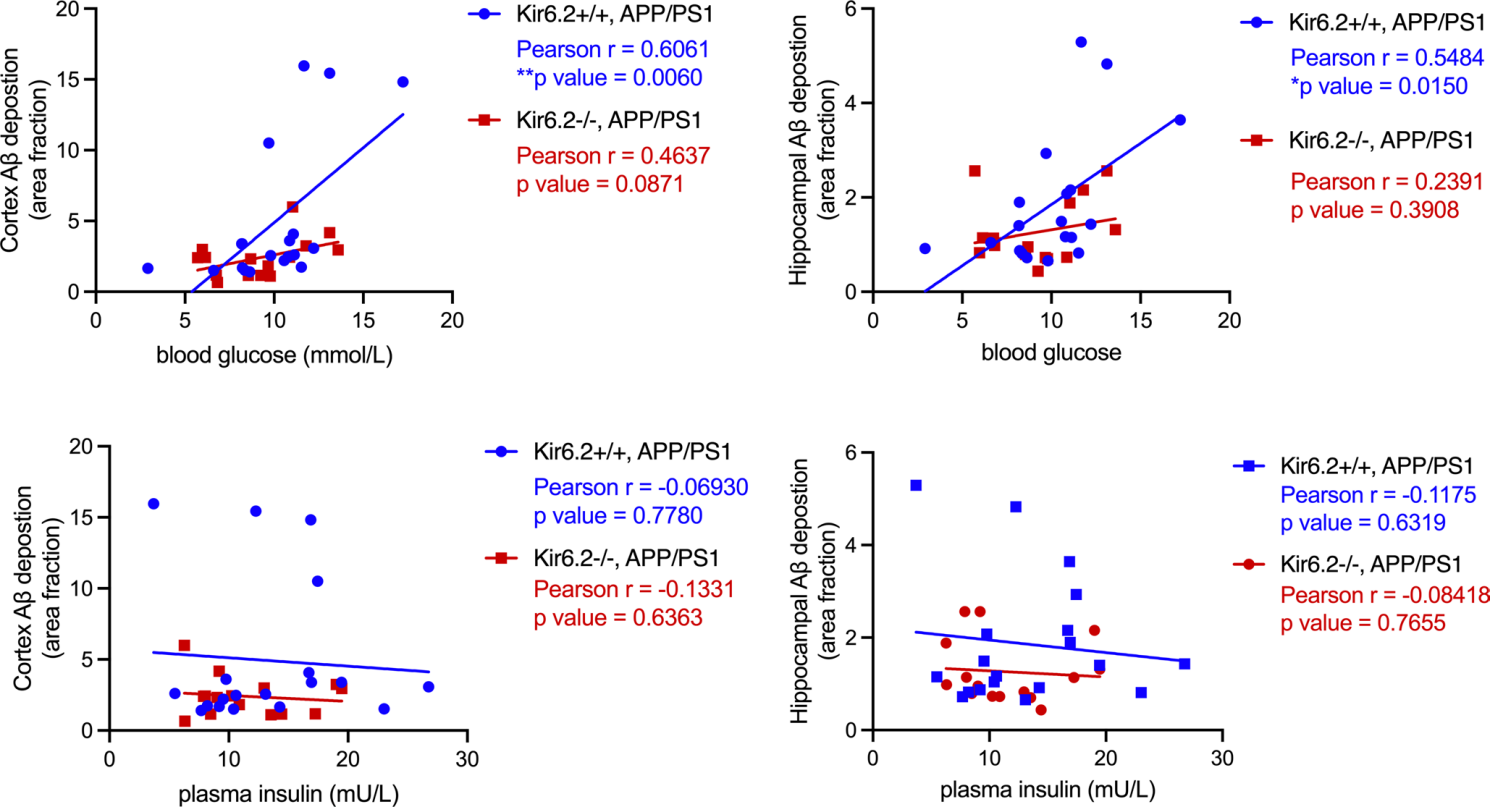
K_ATP_ channels couple changes in blood glucose levels with Aβ pathology. In Kir6.2^+/+^ APP/PS1 mice, there is a significant, positive correlation between blood glucose levels and Aβ deposition in both the cortex (Pearson’s *r* = 0.6061, *P* = 0.006) and hippocampus when mice are on a regular or high-sugar diet (Pearson’s *r* = 0.5484, *P* = 0.0150). Conversely, in Kir6.2^–/–^ APP/PS1 mice, no correlation exists between blood glucose levels and Aβ deposition in either the cortex or hippocampus. Plasma insulin levels were not correlated with Aβ deposition in any condition. All data represented as means ± SEM. *P < 0.05, **P < 0.01.

Next, we explored how changes in peripheral metabolism correlated with changes in Aβ-related pathology in Kir6.2^+/+^ APP/PS1 and Kir6.2^–/–^ APP/PS1 mice (Figure 8). In Kir6.2^+/+^ APP/PS1 mice, peripheral blood glucose levels had a modest, positive correlation with Aβ deposition in both the cortex (Pearson *r* = 0.6061, *P* = 0.0060) and hippocampus (Pearson *r* = 0.5484, *P* = 0.0150). In APP/PS1 mice lacking Kir6.2, no significant correlations persisted between blood glucose levels and Aβ pathology. We also explored whether plasma insulin, another metabolite implicated in AD pathogenesis, correlated with Aβ pathology; however, no correlation between insulin and Aβ existed in either genotype or diet. This suggests that increased blood glucose levels are sufficient to drive Aβ pathology and Kir6.2-K_ATP_ channels are necessary to do so.

### K_ATP_ channel activity is necessary for hyperglycemia-dependent release of astrocytic lactate.

**Figure 9:**
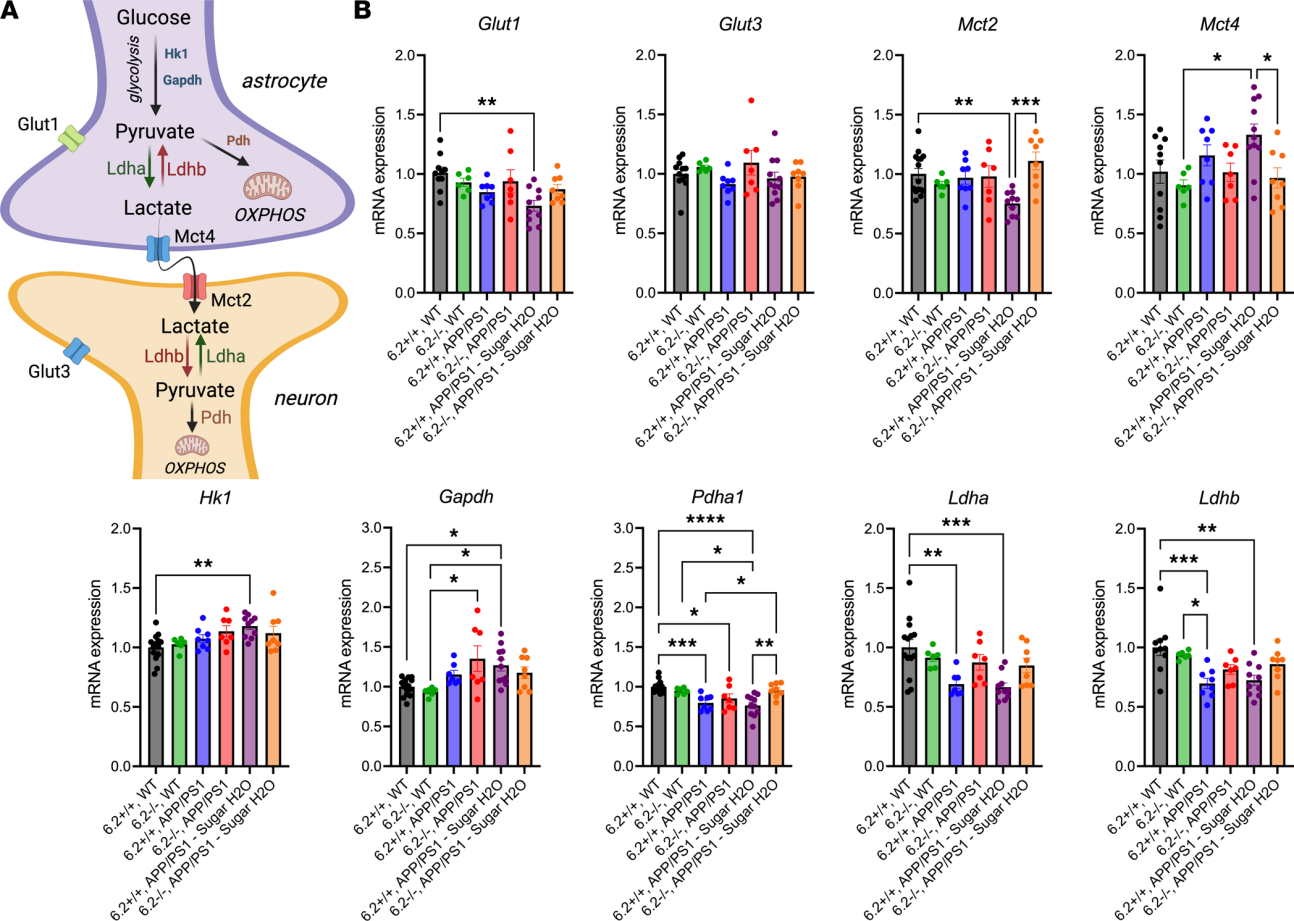
Alterations in lactate metabolism and transport because of the interaction among Aβ, K_ATP_ channels, and a high-sucrose diet. (**A**) Simplified schematic of glucose uptake, lactate production, and lactate transport compartmentalized in astrocytes and neurons. Astrocytes take up glucose via *Glut1* and process it glycolytically (e.g., *Hk1* and *Gapdh*). Pyruvate is converted to lactate via *Ldha* and released extracellularly via *Mct4*. Lactate uptake into neurons is mediated by *Mct2*, where it is converted back to pyruvate via *Ldhb* for use in mitochondrial respiration and oxidative phosphorylation. (**B**) Female Kir6.2^+/+^ APP/PS1 mice on a high-sugar diet had decreased *Glut1* and *Mct2* expression compared with WT, suggesting decreased glucose uptake in astrocytes and decreased lactate uptake in neurons, while displaying increased *Hk1* expression, suggesting increased glycolysis. Kir6.2^–/–^ APP/PS1 mice on a high-sugar diet had an opposing phenotype, where *Mct2* expression increased, *Pdha1* increased, and *Mct4* decreased, suggesting less astrocytic lactate release, conserved neuronal lactate uptake, and preserved mitochondrial respiration. Kir6.2^+/+^ APP/PS1 mice alone displayed decreased *Pdha1*, *Ldha*, and *Ldhb* expression, which was not mirrored in Kir6.2^–/–^ APP/PS1 mice. All relationships were analyzed via 1-way ANOVA with Tukey’s post hoc analysis (*n* = 7–11). All data were analyzed via 1-way ANOVA and represented as means ± SEM. *P < 0.05, **P < 0.01, ***P < 0.001, ****P < 0.0001.

Our data suggest that K_ATP_ channels are necessary for glucose-dependent increases in lactate and Aβ. Therefore, we explored how deletion of Kir6.2-K_ATP_ channels influenced the metabolism and transport of glucose and lactate. According to the astrocyte neuron lactate shuttle hypothesis, glucose uptake, lactate production, and lactate transport are compartmentalized in astrocytes and neurons (44). Astrocytes take up glucose from the bloodstream via glucose transporter 1 (GLUT1) and process it glycolytically via hexokinase 1 (HK1) and GAPDH. Pyruvate is then converted to lactate via lactate dehydrogenase A (LDHA) and released extracellularly via monocarboxylate transporter 4 (MCT4). Lactate uptake into neurons is mediated by monocarboxylate transporter 2 (MCT2), where it is converted back to pyruvate via lactate dehydrogenase B (LDHB) for use in mitochondrial respiration and oxidative phosphorylation via pyruvate dehydrogenase (PDH; Figure 9A). Therefore, we explored whether components of this pathway (*Glut1*, *Glut3*, *Ldha*, *Ldhb*, *Mct2*, *Mct4*, *Hk1*, *Gapdh*, and *Pdha1*) were altered by Aβ pathology, K_ATP_ channel activity, and high-sucrose diet in APP/PS1 mice. Decreased *Pdha1*, *Ldha*, and *Ldhb* expression was observed in Kir6.2^+/+^ APP/PS1 mice, an effect that was not observed in Kir6.2^–/–^ APP/PS1 mice (Figure 9B). High-sugar diet led to decreased *Glut1* and *Mct2* expression in Kir6.2^+/+^ APP/PS1 mice, suggesting decreased glucose uptake in astrocytes and decreased lactate uptake in neurons. High-sugar diet also increased *Hk1* expression in Kir6.2^+/+^ APP/PS1 brains, suggesting increased glycolysis. Conversely, high-sugar diet had an opposing phenotype in Kir6.2^–/–^ APP/PS1 brains, in which *Mct2* and *Pdha1* expression increased, but *Mct4* decreased, suggesting a shift from nonoxidative glucose metabolism to oxidative metabolism. Together, these data suggest that astrocytic production and release of lactate is modulated by Kir6.2-dependent K_ATP_ channels and is important for Aβ release and aggregation.

## Discussion

K_ATP_ channels act as metabolic sensors to modulate a wide array of physiological functions. Their function has been meticulously detailed in pancreatic insulin release (45) and cardiovascular regulation (46–48), and to a lesser extent, in cerebral metabolism (29, 49, 50), neuronal excitability, and Aβ release (12). This study builds upon our previous studies suggesting that hyperglycemia directly influences neuronal Aβ release and offers a mechanism through which hyperglycemia increases Aβ production and deposition. We verified previous findings that CNS hyperglycemia results in Aβ release and showed that these effects are mediated specifically through Kir6.2-K_ATP_ channels.

Our previous data showed that acute hyperglycemia is sufficient to cause increased Aβ release (12), and the goal of this work was to further elucidate the role of Kir6.2-containing K_ATP_ channels in this process. Under acute hyperglycemia, Kir6.2^+/+^ APP/PS1 mice released more Aβ into the hippocampal ISF compared with Kir6.2^–/–^ APP/PS1, demonstrating a relationship between Kir6.2, ISF glucose, and ISF Aβ. Regression analysis of ISF Aβ levels during hyperglycemia showed strong, positive correlations between ISF Aβ, glucose, and lactate levels, affirming a metabolic influence on Aβ release (12, 51–54). Deletion of neuronal K_ATP_ channel activity abolished these relationships in both acute and chronic paradigms, demonstrating K_ATP_ channels linked changes in cerebral metabolism with Aβ production and aggregation.

To further analyze this relationship, we utilized standard APP/PS1 mice with intact K_ATP_ channels, as well as APP/PS1 mice lacking either Kir6.2 (primarily expressed on neurons) or Kir6.1 (primarily expressed on pericytes and endothelial cells) treated with a nonspecific K_ATP_ channel antagonist, glibenclamide. Glibenclamide works to reduce K_ATP_ channel–dependent K^+^ efflux, resulting in a higher resting membrane potential, making affected cells more excitable (33, 45, 48, 49). Since Aβ is released from neurons in an activity-dependent manner, cells that are more readily excited will release more Aβ (12, 39, 55, 56). As such, both Kir6.2^+/+^ APP/PS1 and Kir6.1^–/–^ APP/PS1 mice treated with glibenclamide released more Aβ than Kir6.2^–/–^ APP/PS1 mice. These data demonstrate that Kir6.2- K_ATP_ channels facilitate Aβ release from neurons, not Kir6.1 containing K_ATP_ channels. This is most likely due to Kir6.2- K_ATP_ channels’ localization to neurons and their impact on neuronal excitability and Aβ. These data demonstrate that Kir6.2-containing K_ATP_ channels act as metabolic sensors in the brain and regulate activity-dependent Aβ release and aggregation.

Changes in energy availability through acute hyperglycemic clamps or chronic sugar overconsumption elucidate the complex role K_ATP_ channels play in metabolism and their relationship to AD pathogenesis. Interestingly, Aβ deposition in Kir6.2^+/+^ APP/PS1 and Kir6.2^–/–^ APP/PS1 mice did not differ at baseline, only in response to acute hyperglycemia and chronic sugar H_2_O exposure. In general, neuronal K_ATP_ channel deletion should tend to depolarize excitatory neurons, leading to hyperexcitability. This, in turn, should increase Aβ production and amyloid plaque formation (16–18, 39). However, our results suggest that this is not the case and that Kir6.2^–/–^ mice do not display increased brain hyperexcitability following genetic knockout at baseline. This leads to 3 potential explanations. One is that these animals develop a compensatory mechanism in which other inward-rectifying channels (e.g., Kir6.1-containing K_ATP_ channels) increase expression to compensate for the lack of Kir6.2 channels and prevent hyperexcitability. However, our experiments with direct CNS infusions of the K_ATP_ channel antagonist, glibenclamide, into Kir6.1^–/–^ mice disproves this explanation. An additional explanation, partially supported by our results, is due to the expression of Kir6.2-containing K_ATP_ channels on inhibitory neurons. In many circuits, inhibitory interneurons act as a brake to prevent overexcitation and seizure activity (57, 58). Our results show that while Kir6.2 expression is primarily localized to excitatory neurons, it is also highly expressed on inhibitory neurons. It is unclear how the expression of these channels contributes to the excitatory-inhibitory balance within the brain and what the implications are for Aβ pathology. If these channels are deleted across multiple neuronal populations, then we can deduce that while there may be increased excitability of excitatory neurons, the concurrent increased excitability of inhibitory neurons acts as a brake for this excitation and cancels out the net effect of hyperactivity. This is supported by previous studies that show 1) K_ATP_ channels are located in high density on GABAergic interneurons in multiple brain regions, specifically the hippocampus and substantia nigra, and 2) K_ATP_ channels seem to be involved in the excitation/inhibition balance within circuits (59–62). Thus, a net increase in excitability within a circuit leads to GABAergic interneuron inhibition of glutamatergic hyperexcitability. Additional studies need to explore the role of Kir6.2-containing K_ATP_ channels in excitatory/inhibitory balance in AD to tease this out further. Last, it is also important to acknowledge that Kir6.2^–/–^ mice are a global knockout, and Kir6.2-expressing K_ATP_ channels are found in the peripheral tissues, including striated muscle, which could alter glucose handling. While this is certainly a consideration, in vivo microdialysis allowed for the direct measurement of brain glucose levels during a hyperglycemic clamp, and the levels were comparable between Kir6.2^–/–^ APP/PS1 and Kir6.2^+/+^ APP/PS1 mice, making peripheral modulation of glucose handling less of a concern. Additional studies in conditional knockouts that limit cell type deletion of K_ATP_ channels are necessary to explore these relationships further.

We found no evidence of impaired glucose transport into the brain between Kir6.2^+/+^ APP/PS1 and Kir6.2^–/–^ APP/PS1 mice (Figure 3, A and B), which is important for 2 reasons. First, these studies reinforce the idea that glucose uptake into the brain is largely insulin independent. Since comparable levels of ISF glucose were attained in both the Kir6.2^+/+^ APP/PS1 and Kir6.2^–/–^ APP/PS1 brains with a hyperglycemic clamp, but the Kir6.2^–/–^ APP/PS1 mice did not have a compensatory increase in blood insulin levels, this demonstrates that glucose transport from the blood to the brain is not dependent on or altered by plasma insulin levels. Second, it demonstrates that Kir6.2-K_ATP_ channels are not necessary for glucose transport into the brain. These data coincide with transcriptomic data that show Kir6.2 (KCNJ11/*Kcnj11*) is predominantly expressed on neurons and only minimally expressed on astrocytes, pericytes, and endothelial cells (63). Since endothelial cells, pericytes, and astrocytes are responsible for the initial uptake of glucose from the blood to the brain (64), it is unlikely that the absence of Kir6.2- K_ATP_ channels would affect energy availability in the brain.

While demonstrating a mechanistic relationship between Kir6.2-K_ATP_ channels, glucose metabolism, and Aβ release, our studies also show a potentially unique relationship between K_ATP_ channel activity and lactate production, which may be a necessary step for extracellular Aβ release and subsequent aggregation. It is well documented that ISF and CSF lactate levels display a strong correlation with ISF and CSF levels of Aβ and tau, the pathological hallmarks of AD (9, 12, 14, 26, 39, 65, 66). ISF lactate levels covary with ISF Aβ and ISF tau across the circadian day and sleep/wake cycles, where ISF Aβ, tau, and lactate increase during periods of sleep deprivation (9, 14). During increased periods of neuronal activity, ISF lactate rises and is strongly correlated with increased ISF Aβ (39). In the human brain, aerobic glycolysis, a process where excess glucose is used for lactate production and not oxidative phosphorylation, is a biomarker of brain regions vulnerable to amyloid and tau deposition (67–69). Thus, extracellular lactate levels, at minimum, covary with Aβ and tau and may be more likely to play a role in Aβ and tau aggregation. Our data suggest that K_ATP_ channel activity couples glucose and lactate levels, representing an opportunity to intervene therapeutically and modulate Aβ and tau levels. Our studies also suggest that targeting lactate might be important for reducing Aβ, and perhaps tau, production and aggregation.

We also aimed to discern how a mild metabolic insult via chronic exposure to high-sucrose diet affects AD-related pathology in APP/PS1 mice with or without Kir6.2-dependent K_ATP_ channels. It is well established that T2DM and metabolic syndrome (MetS) are strong risk factors for the development of AD (70–72), but this study reinforces the idea that a subtle change in glucose homeostasis, in the absence of T2DM, MetS, or hyperinsulinemia, is itself sufficient to alter APP processing, Aβ production, and AD-related pathology (12, 54, 73–76). It is important to note that this paradigm was not intended to bring about an overt diabetic or obese phenotype, but rather to analyze how changes in glucose metabolism and energy abundance might alter the delicate balance of cerebral metabolism and drive AD-related pathology.

In the present study, we found that chronic sucrose diet in mice with intact Kir6.2-K_ATP_ channels increased Aβ plaque deposition, ISF Aβ levels, and CTF-β expression. These data offer a stepwise mechanism to demonstrate how Kir6.2-K_ATP_ channels facilitate increases in Aβ plaque deposition in response to glucose dyshomeostasis: 1) Hyperglycemia causes aberrant metabolism that is mediated through the presence of Kir6.2-K_ATP_ channels. 2) This change in neuronal metabolism causes the amyloidogenic processing of APP, resulting in higher CTF-β and Aβ generation (77). 3) Increased stimulation of hippocampal neurons via hyperglycemia and Kir6.2-K_ATP_ channels results in increased Aβ release into ISF. 4) Over time, increased concentration of extracellular Aβ aggregates into extracellular amyloid plaques in a concentration-dependent manner.

Last, this study demonstrates a potentially novel mechanism by which individuals with prediabetes or T2DM may be at an increased risk to develop AD. It demonstrates that increased sugar intake, which drives elevations in blood glucose, are sufficient to drive Aβ pathology, independent of T2DM, obesity, and MetS. Importantly, it also suggests that either pharmacological modulation of Kir6.2-K_ATP_ channels or dietary modifications that include low-carbohydrate diets hold therapeutic promise for patients with diabetes and prediabetes to help reduce the risk of developing AD.

## Methods

### Gene expression alterations in AD brains.

To determine whether K_ATP_ channel genes were differentially expressed in AD brain tissue, we analyzed gene expression of *KCNJ11* and *ABCC8* in 2 publicly available data sets. First, we analyzed gene expression data from the Mayo RNA-Seq study from the AMP-AD portal: the temporal cortex of 80 control, 82 AD, and 29 pathologic aging brains (syn3163039; syn5550404). Multivariable linear regression analyses were performed using conditional quantile–normalized gene expression measures and including age at death, sex, RNA integrity number, brain tissue source, and flowcell as biological and technical covariates (78). Second, we used Mathys et al. (syn18485175) (35) single-nuclei transcriptomics (snRNA-Seq) analysis of AD brains to determine the cell type–specific expression of *KCNJ11* and *ABCC8* in postmortem human prefrontal cortex across the AD continuum (*n* = 48) from the Religious Order Study or the Rush Memory and Aging Project study (79). Individuals had either “no pathology” (*N* = 24) or high levels of Aβ pathology and other AD-associated pathological changes (*N* = 24). In the Aβ-positive group, the participants were further characterized as “early pathology,” suggesting a milder presentation of disease, or “late pathology,” suggesting a more advanced stage of disease. Individuals were balanced between sexes, age, and years of education. We analyzed 80,660 droplet-based snRNA-Seq profiles and assessed differential expression. Methodology used was described extensively before (35). Data were filtered per Mathys et al. (35) and clustered using the SCANPY package in Python (80). The Leiden algorithm was applied to identify cell clusters, and a UMAP algorithm was used for dimension reduction and visualization of clustering results (80).

### APPswe/PS1ΔE9 and Kir6.2^–/–^ mice.

APPswe/PS1ΔE9 mice on a B6C3 [B6;C3-Tg(APPswe, PSEN1dE9)85Dbo/mmjax] background (APP/PS1; from Jackson Labs and bred in-house; ref. 36) were crossed to Kir6.2^–/–^ (C57BL6/J) (33) or Kir6.1^–/–^ (C57BL6/J) (81) (both gifts from Washington University School of Medicine in St. Louis and bred in-house) for these studies. To generate APP/PS1 mice with homozygous knockout (^–/–^) for Kir6.2, we bred Kir6.2^–/–^ mice with APP/PS1 mice, generating Kir6.2^+/+^ APP/PS1 and Kir6.2^–/–^ APP/PS1 mice for the acute and chronic experiments. For reverse microdialysis experiments, we also generated mice homozygous knockout (^–/–^) for Kir6.1 crossed to APP/PS1 mice, generating Kir6.1^–/–^ APP/PS1 mice. Mice were given food and water ad libitum and maintained on a 12-hour light/12-hour dark cycle. All procedures were carried out in accordance with an approved IACUC protocol from Washington University School of Medicine or Wake Forest School of Medicine.

### In vivo microdialysis and hyperglycemic clamps.

Five days prior to glucose clamps (82), 3-month-old Kir6.2^+/+^ APP/PS1 and Kir6.2^–/–^ APP/PS1 mice (*n* = 7–8 mice/group) were anesthetized via isoflurane and tapered catheters (MRE025 tubing, Braintree Scientific) inserted into the jugular vein and the femoral artery and sutured into place. The catheter lines were filled with polyvinylpyrrolidone (PVP), the ends were double knotted, and a suture was affixed to the ends. A small incision was made between the scapulae and the lines tunneled to this area for externalization prior to glucose clamps. Two days prior to glucose clamps, guide cannulas (BR-style, Bioanalytical Systems) were stereotaxically implanted into the hippocampus (from bregma, anterior/posterior: –3.1 mm, medial/lateral: –2.5 mm, dorsal/ventral: –1.2 mm at 12˚ angle) and secured into place with dental cement. Cresyl violet staining was used to validate probe placement postmortem. One day prior to glucose clamps, the mice were transferred to Raturn sampling cages (Bioanalytical Systems), and microdialysis probes (2 mm; 38 kDa molecular weight cutoff; BR-style, Bioanalytical Systems) were inserted into the guide cannula, connected to a syringe pump, and infused with artificial cerebrospinal fluid (1.3 mM CaCl_2_, 1.2 mM MgSO_4_, 3 mM KCl, 0.4 mM KH_2_PO_4_, 25 mM NaHCO_3_, and 122 mM NaCl; pH = 7.35) at a flow rate of 1 μL/min. At this time, the jugular and femoral lines were externalized and the PVP was removed. The femoral and jugular lines were flushed, connected to a syringe pump, and slowly infused with 0.9% sodium chloride at a flow rate of 1 μL/min overnight to prevent clots. Hourly collection of hippocampal ISF began. The following morning, mice were fasted 4–5 hours prior to and during the 4-hour glucose clamps. For the duration of the clamp, the jugular vein was infused with a 12.5% dextrose solution in PBS (control mice received PBS alone) at a variable flow rate. Every 10 minutes, blood was sampled via the femoral artery and blood glucose concentration assessed using a handheld glucometer (Contour, Bayer). The concentration of blood glucose was targeted to 150–200 mg/dL, and the flow rate of the dextrose solution was adjusted accordingly. After the 4-hour clamp, the dextrose solution was stopped, the lines were flushed, euglycemia was restored, and food was returned to the bowls. Hourly ISF collection continued for the duration of the clamp and for 10–15 hours postclamp.

### Glucose tolerance test.

Glucose tolerance test was performed as previously described (83). Briefly, mice were fasted for 4 hours, and 2.0 g/kg glucose was administered via i.p injection. Blood samples were taken from tail veins, and blood glucose was measured at baseline, 15, 30, 45, 60, 90, and 120 minutes from glucose injection using a handheld glucometer (Bound Tree Medical Precision XTRA Glucometer; Thermo Fisher Scientific).

### Glibenclamide administration via reverse microdialysis.

Guide cannula implantation and in vivo microdialysis were performed as described above. Glibenclamide (100 μM; MilliporeSigma) was infused directly into the hippocampus of Kir6.2^+/+^ APP/PS1 mice, Kir6.2^–/–^ APP/PS1 mice, and Kir6.1^–/–^ APP/PS1 mice (*n* = 4–7) via reverse microdialysis for 3 hours. Changes in ISF glucose, lactate, and Aβ were quantified as described below. Statistical significance was determined using a 1-way ANOVA and Dunnett’s multiple-comparison post hoc test. Data are represented by means ± SEM.

### Aβ_1–x_ ELISA.

ISF samples from Kir6.2^+/+^ APP/PS1 mice, Kir6.2^–/–^ APP/PS1 mice, and Kir6.1^–/–^ APP/PS1 mice (*n* = 7–8/group) collected from in vivo microdialysis experiments were analyzed for Aβ_1–x_ using sandwich ELISAs as previously described (14, 39). Briefly, Aβ_1–x_ was quantified using a monoclonal capture antibody targeted against Aβ13–28 (m266) and a biotinylated detection antibody targeted against Aβ1–5 (3D6), both gifts from Ron DeMattos, Eli Lilly and Co., Indianapolis, Indiana, USA. After incubation with streptavidin-poly-HRP-20 (Thermo Fisher Scientific), the assay was developed using Super Slow TMB (MilliporeSigma), and the plates were read on a Bio-Tek Synergy 2 plate reader (Agilent) at 650 nm. Statistical significance was determined using a 2-tailed, unpaired Student’s *t* test. Data are represented by means ± SEM.

### Chronic high-sucrose diet.

Female Kir6.2^+/+^ APP/PS1 mice and Kir6.2^–/–^ APP/PS1 mice were randomized to either regular drinking water group or high-sugar (104 mM glucose, 128 mM fructose) drinking water group (*n* = 8–11 mice/group) at 3 months of age. Mice were subjected to either condition for 6 months, where body weight and blood glucose were monitored at baseline, 6 months of age, and 9 months of age in all animals. At 9 months of age, mice were sacrificed and processed for several biochemical, metabolic, and AD-related pathology analyses.

### Tissue collection.

At the completion of each experiment, mice were deeply anesthetized using isoflurane, received a cardiac puncture to collect blood, and were transcardially perfused with heparinized 1× Dulbecco’s PBS (DPBS). Each brain was bisected into left and right hemispheres. One hemisphere was dissected to isolate specific brain regions for biochemical and molecular biology assays, and the other hemisphere was fixed in 4% paraformaldehyde (PFA) in 1× DPBS for 48 hours at 4°C. PFA-fixed hemispheres were then transferred to 30% sucrose in 1× DPBS for cryoprotection and stored at 4°C until further tissue processing. Brains were sectioned on a freezing microtome (Leica) at 50 μm sections.

Blood collected from cardiac puncture during sacrifice was transferred to a microcentrifuge tube coated in EDTA to prevent coagulation. Blood was spun down at 2,000*g* for 10 minutes at 4°C to isolate plasma from the whole blood. The plasma supernatant was then removed, transferred to a clean tube, and stored at –80°C until use.

### Insulin ELISA.

Plasma insulin levels of 9-month-old animals were measured via mouse ultra-sensitive insulin ELISA (ALPCO) according to manufacturer’s protocol. Briefly, manufacturer-provided standard curve samples (0, 0.188, 0.5, 1.25, 3.75, and 6.9 ng/mL) were loaded in triplicate. Duplicate 5 μL samples of plasma were loaded into a 96-well plate that was precoated with a capture insulin antibody. A total of 75 μL conjugated substrate was added to each well and mixed for 2 hours on an orbital shaker at 450 rpm. Wells were then washed 6 times with 1× wash buffer. Then 100 μL of TMB substrate was added to each well and mixed for 30 minutes at room temperature. A total of 100 μL of stop solution was added to each well and gently mixed prior to reading. The plate was then immediately read at 450 nm. The standard curve was constructed using a 5-parameter logistic curve, and sample optical densities were compared with the standard curve to determine the plasma insulin concentration. Plasma insulin levels were analyzed via 1-way ANOVA and Tukey’s post hoc tests.

### Glucose and lactate measures.

Glucose and lactate measurements from blood and hippocampal ISF were quantified using an analyzer (YSI 2900) using glucose- and lactate-oxidase method per the manufacturer’s instructions as previously described (12). The ratio of plasma glucose to lactate was also calculated by plasma glucose/plasma lactate. Plasma levels of glucose, lactate, and glucose/lactate were analyzed via 1-way ANOVA and Tukey’s post hoc tests. Significance was determined at *P* < 0.05, while a trend was *P* < 0.1. Data are represented by means ± SEM.

### Immunohistochemical staining of brains using anti-Aβ antibodies.

Serial sections (300 μm apart) through the anterior-posterior aspect of the hippocampus were immunostained for Aβ deposition (anti-HJ3.4B, a gift from Washington University in St. Louis, St. Louis, Missouri, USA). Freely floating sections were stained using a biotinylated HJ3.4 antibody (anti–Aβ1–13, mouse monoclonal antibody, a gift from Washington University in St. Louis, St. Louis, Missouri, USA) and developed using a Vectastain ABC Elite kit (Vector Laboratories) and DAB reaction. Stained brain sections were imaged using a NanoZoomer slide scanner (Hamamatsu Photonics), and the percentage area occupied by HJ3.4 was quantified by a masked researcher as previously described (5, 6). Two-way repeated measures ANOVAs were used to analyze differences of between-subject factors (genotype and diet), and post hoc analyses (Tukey’s test for multiple comparisons) were performed for assessing specific group comparisons.

### Western blotting.

Western blot analysis was used to measure levels of APP and the CTFs CTF-α and CTF-β produced from cleavage of APP by α-secretases or β- and γ-secretases, respectively. Briefly, hippocampi were homogenized in 1× cell lysis buffer (Cell Signaling Technology) supplemented with protease inhibitor cocktail (Roche), 1 mM PMSF (Cell Signaling Technology), 1 mM DTT (MilliporeSigma), and a phosphatase inhibitor cocktail (MilliporeSigma) using a probe sonicator at 30% amplitude, 1-second pulse with a 5-second delay 5 times, while on ice. Tissue homogenates were then spun down at 10,000*g* for 10 minutes at 4°C, and the supernatant was removed and used for immunoblotting. Protein concentrations were analyzed using BCA Protein Assay Kit (Pierce). We ran 25 μg of protein via SDS-PAGE in 10% Tris-tricine gels using Bio-Rad protean mini and then rapidly transferred to PVDF membranes using Bio-Rad semi-dry. Membranes were subsequently blocked using 5% BSA in 1× TBS-Tween for 1 hour and then incubated with either 1° (1:1,000) APP and CTFs (catalog 51-2700, Life Technologies) or the loading control (1:50,000) β-actin (MAB1501, MilliporeSigma) overnight at 4°C. A 2° antibody conjugated with HRP specific to either (1:3,000) goat anti-rabbit (7074, Cell Signaling Technology) or (1:3,000) goat anti-mouse (7076, Cell Signaling Technology) were incubated for 1 hour at room temperature. Chemiluminescence was measured using ECL (MilliporeSigma). All images were quantified using ImageJ (NIH), normalized to β-actin, and analyzed via 2-way repeated measures ANOVAs to analyze differences of between-subject factors (genotype and diet). Post hoc analyses (Tukey’s test for multiple comparisons) were performed to determine specific group comparisons.

### RNA extraction.

Frozen cortex was homogenized in QIAzol (QIAGEN) using a sterile, nuclease-free handheld homogenizer (Bel-art) for approximately 30 seconds on ice. Samples then sat at room temperature for 5 minutes and then were mixed vigorously with chloroform at 1:5 ratio (chloroform/QIAzol) and allowed to sit for 10 minutes at room temperature. Sample mixtures were then spun down at 12,000*g* for 10 minutes at 4°C. The aqueous layer of RNA was then carefully removed, gently mixed with 70% EtOH/original volume of QIAzol (1:1), and run through an RNeasy spin column (QIAGEN) according to manufacturer’s protocol.

### qPCR.

A total of 1 μg of RNA was then converted to cDNA using High-Capacity cDNA Reverse Transcription kit (Applied Biosystems) according to manufacturer’s protocol. cDNA was then used to run qPCR for several target genes: Kcnj8 (Mm00434620_m1, Life Technologies), Kcnj11 (Mm04243639_s1, Life Technologies), Abcc8 (Mm00803450_m1, Life Technologies), Abcc9 (313340540, IDT), Ldha (Mm01612132_g1, Life Technologies), Ldhb (Mm01267402_m1, Life Technologies), Glut1 (316054177, IDT), Glut3 (Mm00441483_m1), Mct2 (Mm00441442_m1, Life Technologies), and Mct4 (Mm0046102_m1, Life Technologies) using Rn-18s (Mm03928990_g1, Life Technologies) as a loading control. Briefly, 20 ng of cDNA was loaded per well with TaqMan Fast Advanced Master Mix (Applied Biosystems) along with 0.5 μL of primer for a specific gene of interest and 0.5 μL of Rn-18s primer. qPCR was run using QuantStudio 6 Pro (Applied Biosystems) according to manufacturer’s protocols for TaqMan primers (Life Technologies) and TaqMan Fast Advanced Master Mix. Relative quantities of gene expression were quantified as described (84), and gene expression was statistically analyzed via 1-way ANOVA using GraphPad Prism.

### Statistics.

Statistical analyses using Pearson’s *r* correlation, 2-tailed Student’s *t* test, 1-way ANOVA, and 2-way ANOVA using appropriate post hoc tests were performed for each previously mentioned experiment. *P* values less than 0.05 were considered significant and 0.05 ≤ *P* ≤ 0.10 was considered a trend. All tests were conducted using GraphPad Prism.

### Study approval.

All animal procedures and experiments were performed under guidelines approved by the IACUC at Washington University School of Medicine in St. Louis or Wake Forest School of Medicine.

### Data availability.

All data generated or analyzed during this study are included in this published article.

## Author contributions

SLM, DMH, and MCP conceived of the study. SLM, DMH, MSR, CGN, CMK, JG, and MCP contributed to study design. SLM, JG, MS, EEC, WRM, CMC, JAS, SDK, LJD, JD, NIN, NJC, RJPM, SMD, and DK performed experiments, data analysis, and data interpretation. SLM, JG, CGN, and NJC wrote the manuscript. JG and WRM contributed equally to the manuscript. JG was given first author position because of his role in writing and revising the manuscript. JG, WRM, MCP, MS, SDK, CMC, NJC, LJD, JAS, DK, EEC, RJPM, SMD, JPS, NIN, JD, MSR, DDK, CMK, CGN, DMH, and SLM discussed the results and commented on the manuscript.

## Supplementary Material

Supplemental data

## Version 1 05/02/2023

In-Press Preview

## Version 2 05/22/2023

Electronic publication

## References

[1] Ott A (1999). Diabetes mellitus and the risk of dementia: the Rotterdam study. Neurology.

[2] Biessels GJ (2005). Increased risk of Alzheimer’s disease in type II diabetes: insulin resistance of the brain or insulin-induced amyloid pathology?. Biochem Soc Trans.

[3] Bosco D (2011). Possible implications of insulin resistance and glucose metabolism in Alzheimer’s disease pathogenesis. J Cell Mol Med.

[4] Huang CC (2014). Diabetes mellitus and the risk of Alzheimer’s disease: a nationwide population-based study. PLoS One.

[5] Crane PK (2013). Glucose levels and risk of dementia. N Engl J Med.

[6] Morris JK (2014). Impaired glycemia increases disease progression in mild cognitive impairment. Neurobiol Aging.

[7] Sato N, Morishita R (2014). Brain alterations and clinical symptoms of dementia in diabetes: aβ/tau-dependent and independent mechanisms. Front Endocrinol (lausanne).

[8] Long JM, Holtzman DM (2019). Alzheimer disease: an update on pathobiology and treatment strategies. Cell.

[9] Holth JK (2019). The sleep-wake cycle regulates brain interstitial fluid tau in mice and CSF tau in humans. Science.

[10] Arnold SE (2018). Brain insulin resistance in type 2 diabetes and Alzheimer disease: concepts and conundrums. Nat Rev Neurol.

[11] Stanley M (2016). Changes in insulin and insulin signaling in Alzheimer’s disease: cause or consequence?. J Exp Med.

[12] Macauley SL (2015). Hyperglycemia modulates extracellular amyloid-β concentrations and neuronal activity in vivo. J Clin Invest.

[13] Yamada K (2014). Neuronal activity regulates extracellular tau in vivo. J Exp Med.

[14] Roh JH (2012). Disruption of the sleep-wake cycle and diurnal fluctuation of β-amyloid in mice with Alzheimer’s disease pathology. Sci Transl Med.

[15] Bero AW (2012). Bidirectional relationship between functional connectivity and amyloid-β deposition in mouse brain. J Neurosci.

[16] Verges DK (2011). Opposing synaptic regulation of amyloid-β metabolism by NMDA receptors in vivo. J Neurosci.

[17] Cirrito JR (2005). Synaptic activity regulates interstitial fluid amyloid-beta levels in vivo. Neuron.

[18] Cirrito JR (2003). In vivo assessment of brain interstitial fluid with microdialysis reveals plaque-associated changes in amyloid-beta metabolism and half-life. J Neurosci.

[19] Yamamoto K (2015). Chronic optogenetic activation augments aβ pathology in a mouse model of Alzheimer disease. Cell Rep.

[20] Vossel KA (2017). Epileptic activity in Alzheimer’s disease: causes and clinical relevance. Lancet Neurol.

[21] Vossel KA (2013). Seizures and epileptiform activity in the early stages of Alzheimer disease. JAMA Neurol.

[22] Gureviciene I (2019). Characterization of epileptic spiking associated with brain amyloidosis in APP/PS1 mice. Front Neurol.

[23] Gurevicius K (2013). Short- and long-term habituation of auditory event-related potentials in the rat. F1000Res.

[24] Stanley M (2016). The effects of peripheral and central high insulin on brain insulin signaling and amyloid-β in young and old APP/PS1 mice. J Neurosci.

[25] Kavanagh K (2019). Type-2-diabetes alters CSF but not plasma metabolomic and AD risk profiles in vervet monkeys. Front Neurosci.

[26] Harris RA (2016). Aerobic glycolysis in the frontal cortex correlates with memory performance in wild-type mice but not the APP/PS1 mouse model of cerebral amyloidosis. J Neurosci.

[27] Nichols CG (2006). KATP channels as molecular sensors of cellular metabolism. Nature.

[28] Foster MN, Coetzee WA (2016). KATP channels in the cardiovascular system. Physiol Rev.

[29] Huang CW (2007). Glucose and hippocampal neuronal excitability: role of ATP-sensitive potassium channels. J Neurosci Res.

[30] Tanner GR (2011). Single K ATP channel opening in response to action potential firing in mouse dentate granule neurons. J Neurosci.

[31] Hsu C-C (2011). Incidence of dementia is increased in type 2 diabetes and reduced by the use of sulfonylureas and metformin. J Alzheimers Dis.

[32] Gradman TJ (1993). Verbal learning and/or memory improves with glycemic control in older subjects with non-insulin-dependent diabetes mellitus. J Am Geriatr Soc.

[33] Miki T (1998). Defective insulin secretion and enhanced insulin action in K_ATP_ channel-deficient mice. Proc Natl Acad Sci U S A.

[34] Remedi MS (2006). Hyperinsulinism in mice with heterozygous loss of K_ATP_ channels. Diabetologia.

[35] Mathys H (2019). Single-cell transcriptomic analysis of Alzheimer’s disease. Nature.

[36] Jankowsky JL (2004). Mutant presenilins specifically elevate the levels of the 42 residue beta-amyloid peptide in vivo: evidence for augmentation of a 42-specific gamma secretase. Hum Mol Genet.

[37] Stanley M (2016). The effects of peripheral and central high insulin on brain insulin signaling and amyloid-β in young and old APP/PS1 mice. J Neurosci.

[38] Rocheleau JV (2006). Critical role of gap junction coupled KATP channel activity for regulated insulin secretion. PLoS Biol.

[39] Bero AW (2011). Neuronal activity regulates the regional vulnerability to amyloid-β deposition. Nat Neurosci.

[40] Sotello D (2019). Glucose and lactate levels at admission as predictors of in-hospital mortality. Cureus.

[41] Silva CM (2022). Prognostic value of hyperlactatemia in infected patients admitted to intensive care units: a multicenter study. Rev Bras Ter Intensiva.

[42] Cornford EM (2002). Regional analyses of CNS microdialysate glucose and lactate in seizure patients. Epilepsia.

[43] During MJ (1994). Direct measurement of extracellular lactate in the human hippocampus during spontaneous seizures. J Neurochem.

[44] Pellerin L, Magistretti PJ (1994). Glutamate uptake into astrocytes stimulates aerobic glycolysis: a mechanism coupling neuronal activity to glucose utilization. Proc Natl Acad Sci U S A.

[45] Koster JC (2005). Diabetes and insulin secretion: the ATP-sensitive K^+^ channel (K_ATP_) connection. Diabetes.

[46] McClenaghan C (2020). Glibenclamide reverses cardiovascular abnormalities of Cantu syndrome driven by K ATP channel overactivity. J Clin Invest.

[47] Nichols CG (2006). K ATP channels as molecular sensors of cellular metabolism. Nature.

[48] Nichols CG (2013). KATP channels and cardiovascular disease: suddenly a syndrome. Circ Res.

[49] Tanner GR (2011). Single KATP channel opening in response to action potential firing in mouse dentate granule neurons. J Neurosci.

[50] Nelson PT (2015). ABCC9/SUR2 in the brain: Implications for hippocampal sclerosis of aging and a potential therapeutic target. Ageing Res Rev.

[51] Nagai N (2016). Hyperglycemia enhances the production of amyloid β1-42 in the lenses of Otsuka long-evans tokushima fatty rats, a model of human type 2 diabetes. Invest Ophthalmol Vis Sci.

[52] Yang Y (2013). High glucose promotes Aβ production by inhibiting APP degradation. PLoS One.

[53] Sah SK (2017). Effect of high-fat diet on cognitive impairment in triple-transgenic mice model of Alzheimer’s disease. Biochem Biophys Res Commun.

[54] Thériault P (2016). High fat diet exacerbates Alzheimer’s disease-related pathology in APPswe/PS1 mice. Oncotarget.

[55] Kang J-E (2007). Acute stress increases interstitial fluid amyloid-beta via corticotropin-releasing factor and neuronal activity. Proc Natl Acad Sci U S A.

[56] Cirrito JR (2008). Endocytosis is required for synaptic activity-dependent release of amyloid-beta in vivo. Neuron.

[57] Badawy RA (2012). Epilepsy: ever-changing states of cortical excitability. Neuroscience.

[58] Kirmse K, Zhang C (2022). Principles of GABAergic signaling in developing cortical network dynamics. Cell Rep.

[59] Yamada K (2001). Protective role of ATP-sensitive potassium channels in hypoxia-induced generalized seizure. Science.

[60] Yamada S (2001). Glutamate is not a major conveyer of ATP-sensitive K+ channel-independent glucose action in pancreatic islet beta cell. Endocr J.

[61] Zawar C (1999). Cell-type specific expression of ATP-sensitive potassium channels in the rat hippocampus. J Physiol.

[62] Amoroso S (1990). Glucose, sulfonylureas, and neurotransmitter release: role of ATP-sensitive K^+^ channels. Science.

[63] Zhang Y (2014). An RNA-sequencing transcriptome and splicing database of glia, neurons, and vascular cells of the cerebral cortex. J Neurosci.

[64] Mathiisen TM (2010). The perivascular astroglial sheath provides a complete covering of the brain microvessels: an electron microscopic 3D reconstruction. Glia.

[65] Ju YS (2017). Slow wave sleep disruption increases cerebrospinal fluid amyloid-β levels. Brain.

[66] Blattner MS (2020). Increased cerebrospinal fluid amyloid-β during sleep deprivation in healthy middle-aged adults is not due to stress or circadian disruption. J Alzheimers Dis.

[67] Vaishnavi SN (2010). Regional aerobic glycolysis in the human brain. Proc Natl Acad Sci U S A.

[68] Vlassenko AG (2010). Spatial correlation between brain aerobic glycolysis and amyloid-β (Aβ) deposition. Proc Natl Acad Sci U S A.

[69] Vlassenko AG (2018). Aerobic glycolysis and tau deposition in preclinical Alzheimer’s disease. Neurobiol Aging.

[70] Bharadwaj P (2017). The link between type 2 diabetes and neurodegeneration: roles for amyloid-β, amylin, and Tau proteins. J Alzheimers Dis.

[71] Carr MC, Brunzell JD (2004). Abdominal obesity and dyslipidemia in the metabolic syndrome: importance of type 2 diabetes and familial combined hyperlipidemia in coronary artery disease risk. J Clin Endocrinol Metab.

[72] De la Monte SM, Wands JR (2008). Alzheimer’s disease is type 3 diabetes—evidence reviewed. J Diabetes Sci Technol.

[73] Busquets O (2017). Long-term exposition to a high fat diet favors the appearance of β-amyloid depositions in the brain of C57BL/6J mice. A potential model of sporadic Alzheimer’s disease. Mech Ageing Dev.

[74] Ettcheto M (2016). Evaluation of neuropathological effects of a high-fat diet in a presymptomatic Alzheimer’s disease stage in APP/PS1 mice. J Alzheimers Dis.

[75] Walker JM (2017). Reversal of high fat diet-induced obesity improves glucose tolerance, inflammatory response, β-amyloid accumulation and cognitive decline in the APP/PSEN1 mouse model of Alzheimer’s disease. Neurobiol Dis.

[76] Yoon G (2019). Transcriptomic analysis of high fat diet fed mouse brain cortex. Front Genet.

[77] Chow VW (2010). An overview of APP processing enzymes and products. Neuromolecular Med.

[78] Allen M (2016). Human whole genome genotype and transcriptome data for Alzheimer’s and other neurodegenerative diseases. Sci Data.

[79] Bennett DA (2018). Religious Orders Study and Rush Memory and Aging Project. J Alzheimers Dis.

[80] Wolf FA (2018). SCANPY: large-scale single-cell gene expression data analysis. Genome Biol.

[81] Miki T (2002). Mouse model of Prinzmetal angina by disruption of the inward rectifier Kir6.1. Nat Med.

[82] Berglund ED (2008). Glucose metabolism in vivo in four commonly used inbred mouse strains. Diabetes.

[83] Day SM (2019). Glucagon-like peptide-1 cleavage product improves cognitive function in a mouse model of Down syndrome. eNeuro.

[84] Livak KJ, Schmittgen TD (2001). Analysis of relative gene expression data using real-time quantitative PCR and the 2− ΔΔCT method. Methods.

