# Supplementary material for: KATP channels are necessary for glucose-dependent increases in amyloid-β and Alzheimer’s disease–related pathology
Source: JCI Insight. 2023 May 22;8(10):e162454. doi: 10.1172/jci.insight.162454 (PMC10386887; doi:10.1172/jci.insight.162454)
Supplement: Supplemental data [file jciinsight-8-162454-s021.pdf]

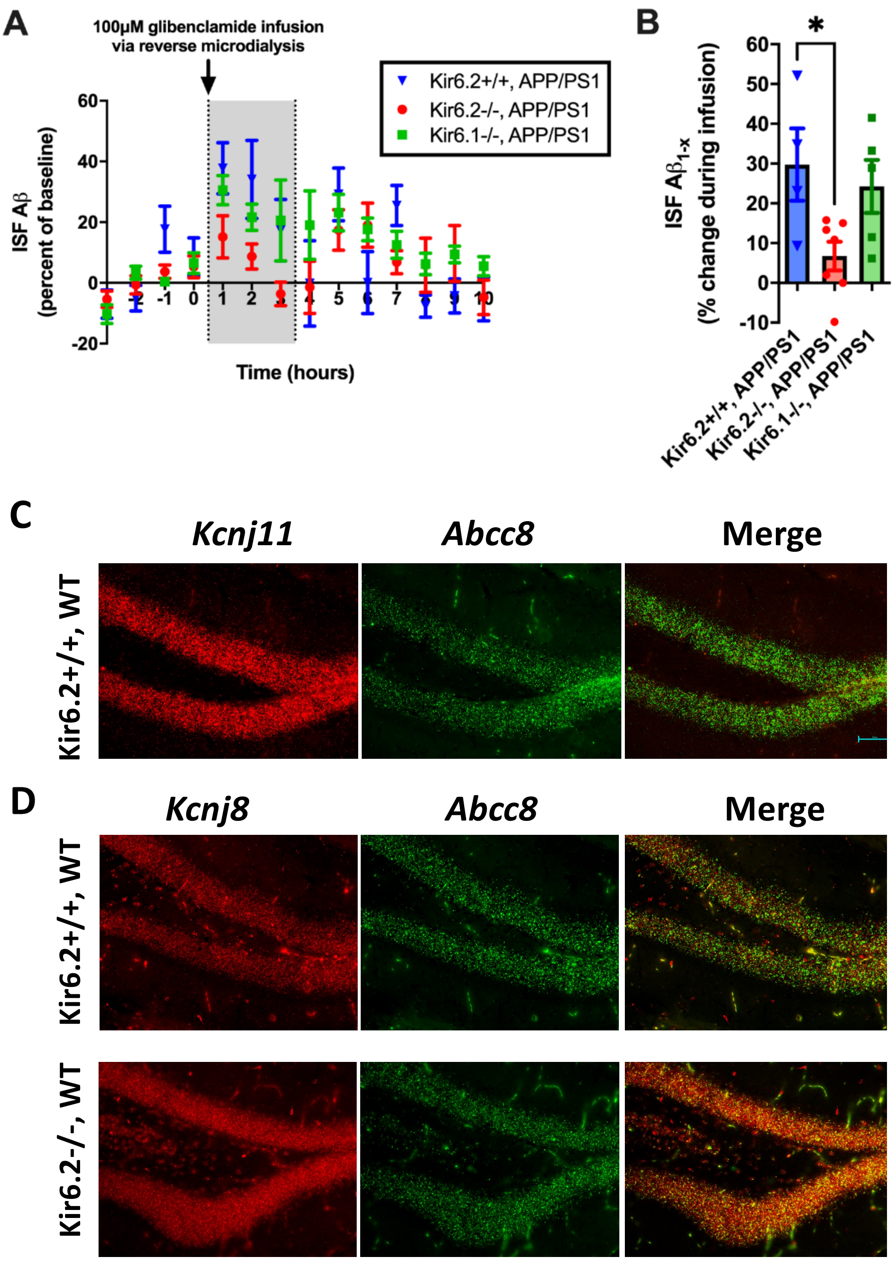

Supplemental Figure 1. Kir6.2-, not Kir6.1-, KATP channels modulate ISF Aβ levels in APP/PS1 mice. (A) 100 µm glibenclamide was infused directly into the hippocampus of male Kir6.2+/+, APP/PS1 (blue triangles), Kir6.1-/-, APP/PS1 (green squares), or Kir6.2-/-, APP/PS1 (red circles) mice (n = 4-7 mice/group). ISF Aβ levels were measured hourly during the 4-hr baseline, 3-hr infusion, and 6-hrs post-infusion (n = 5-7/group). **(B)** During the glibenclamide infusion, ISF Aβ levels rose in Kir6.2+/+,APP/PS1 mice or Kir6.1-/-, APP/PS1 mice by 30% or 24%, respectively. Conversely, ISF Aβ levels rose by only 6% in Kir6.2-/-, APP/PS1 mice which was different than both other groups, suggesting Kir6.2-K_{ATP} channels, not Kir6.1, are necessary for K_{ATP} channel dependent increases in ISF Aβ. ISF Aβ levels were analyzed via one-way ANOVA with Tukey's post hoc analysis. **(C)** RNAscope demonstrates that the subunits Kir6.2 (KCNJ11) and SUR1 (ABCC8) are localized to the dentate gyrus of the hippocampus. **(D)** In the Kir6.2-/- mice, the localization of the Kir6.1 subunit (KCNJ8) expression is similar in Kir6.2-/- and WT, suggesting KCNJ8 does not alter its localization in the absence of KCNJ11. All data represented as means ± SEM. *=p<0.05
